# PChopper: high throughput peptide prediction for MRM/SRM transition design

**DOI:** 10.1186/1471-2105-12-338

**Published:** 2011-08-15

**Authors:** Vackar Afzal, Jeffrey T-J Huang, Abdel Atrih, Daniel J Crowther

**Affiliations:** 1 Translational Medicine Research Collaboration. Dundee, DD1 9SY, UK; 2 College of Life Sciences, University of Dundee, DD1 5EH, UK; 3 Sanofi-Aventis, Industriepark Höchst, 65926 Frankfurt am Main, Germany

## Abstract

**Background:**

The use of selective reaction monitoring (SRM) based LC-MS/MS analysis for the quantification of phosphorylation stoichiometry has been rapidly increasing. At the same time, the number of sites that can be monitored in a single LC-MS/MS experiment is also increasing. The manual processes associated with running these experiments have highlighted the need for computational assistance to quickly design MRM/SRM candidates.

**Results:**

PChopper has been developed to predict peptides that can be produced via enzymatic protein digest; this includes single enzyme digests, and combinations of enzymes. It also allows digests to be simulated in 'batch' mode and can combine information from these simulated digests to suggest the most appropriate enzyme(s) to use. PChopper also allows users to define the characteristic of their target peptides, and can automatically identify phosphorylation sites that may be of interest. Two application end points are available for interacting with the system; the first is a web based graphical tool, and the second is an API endpoint based on HTTP REST.

**Conclusions:**

Service oriented architecture was used to rapidly develop a system that can consume and expose several services. A graphical tool was built to provide an easy to follow workflow that allows scientists to quickly and easily identify the enzymes required to produce multiple peptides in parallel via enzymatic digests in a high throughput manner.

## Background

Selective reaction monitoring-mass spectrometry (SRM-MS) has become a key proteomics technology. It is used in the quantification of post-translational modifications, discrimination of homologous protein isoforms and often as the final step in biomarker discovery. A typical SRM assay consists of two parts, the first involves selecting enzymes that can produce peptides with some target characteristics, and the second involves experimental testing to verify the predictions from the first phase. The manual processes associated with the first phase often makes it prohibitively time-consuming to manually identify the optimal enzyme to give best peptide characteristics and SRM transitions for mass spectrometry, especially if there are multiple protein targets involved. In response to this, a number of software tools have been developed to assist with this process [1-4]. A further in depth review of current software has been performed in [5].

In more complex situations such as quantification of post-translational modifications, there are often multiple target sites on multiple proteins of interest and it is at this point that the limitations of existing software solutions become apparent, and indeed fall short of what is required. In this publication, we shall present PChopper, which has been developed to aid in SRM-assay design with a focus on studies investigating protein phosphorylation stoichiometry, although the tool can be used to support batch SRM-assay design for any study. PChopper is not limited exclusively to trypsin based digests in comparison with most currently available software solutions. PChopper can simulate digests involving a single enzyme, or any combination of two supported enzymes. Each digest can also be parameterised with the target characteristics required of the resultant peptides. Digests can be performed in batch mode, and the output from each digest can be combined into a single dashboard for export.

## Implementation

### Architecture

**Figure 1:**
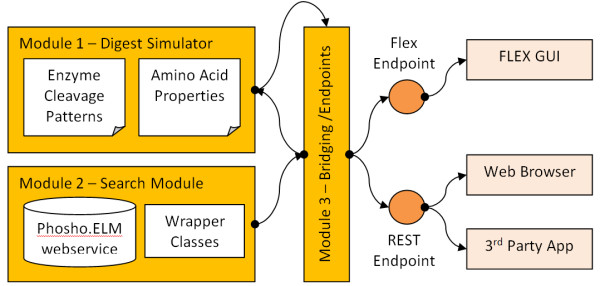
**PChopper architecture**. An overview of the PChopper architecture. Module 3 makes use of modules 1 and 2 to form the core of the application, and exposes two service endpoints.

PChopper utilises a Service Oriented Architecture (SOA) [6] to consume and expose several services. This allows for rapid development since several core services are immediately available with no internal maintenance or development overhead (additional SOA benefits are outlined elsewhere [7,8]). However the use of a service oriented architecture is not without caveats; it creates external system dependencies that PChopper must rely on, but cannot control. Despite this drawback, a service oriented approach was adopted as the benefits outweighed the risks. PChopper also exposes two application endpoints. The first is a graphical user interface that provides an easy to follow workflow for running simulated digests and the second is an API-based programmatic endpoint that allows other developers to make use of the PChopper engine programmatically. Figure 1 provides an overview of the system architecture.

### Workflow

**Figure 2:**
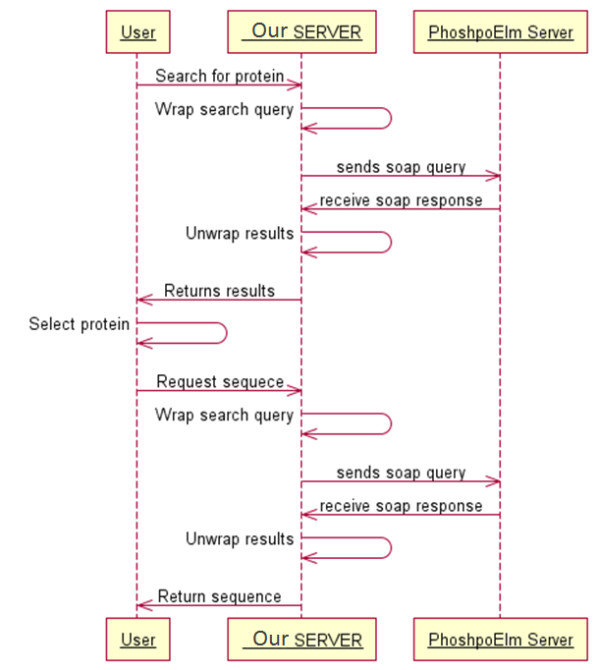
**Webservice communication overview**. A UML Sequence diagram outlining the communication process involved in specifying a protein sequence. The process begins by the user entering a search term for the protein of interest. The PChopper server then wraps this request for submission to the Phosho.ELM web-service; the Phosho.ELM webservice then processes the request and returns the results to the PChopper server where the results are unwrapped and presented back to the user in the form of a selectable list. The user then selects one of the results from the search, and the process of wrapping/unwrapping the initial request and their corresponding results is repeated and the selected sequence is presented back to the user for verification.

**Figure 3:**
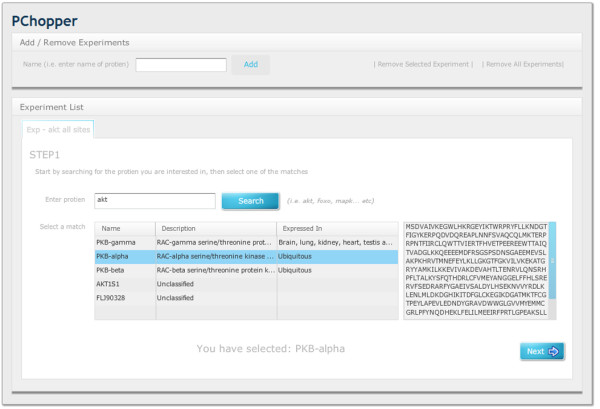
**Workflow Step1**. A user begins by naming the 'experiment'. They then search for the protein of interest and select a result to proceed to the next step in the workflow. In this example the user has searched for AKT and PChopper has performed a fuzzy search and presented the results back to user. In this case AKT has positively identified PKB alpha, beta and gamma in the result list. Selecting one of the results triggers an action which displays the sequence for the selected result. Once the user has made a selection they can progress onto the next stage in the workflow.

**Figure 4:**
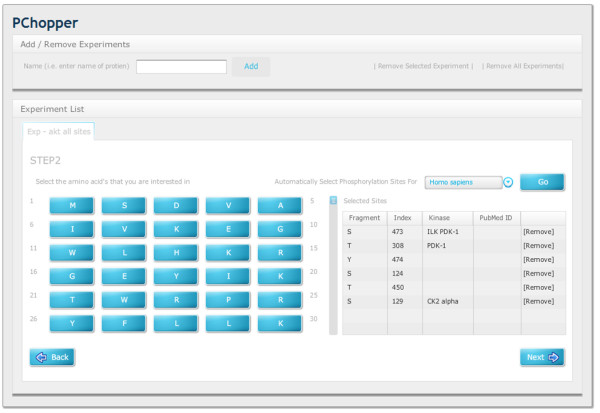
**Workflow Step2**. Users either manually select phosphorylation sites, or they let PChopper select them automatically. Once the user has made their selections they can proceed to the next step in the workflow.

**Figure 5:**
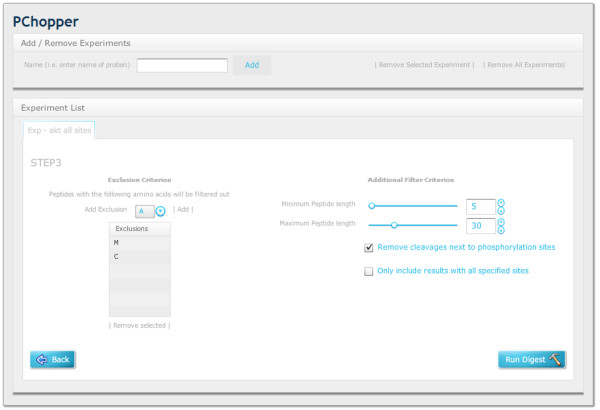
**Workflow Step3**. Users specify the amino acids that they would not like to be included in the resultant peptides (i.e. no M, C due to difficulties with post translational modifications) and the target length of resultant peptide (i.e. between 5 and 30). If there is a phosphorylation site adjacent to an enzyme cleavage site, the cleavage can be missed. This can be simulated by selecting 'Remove cleavages next to phosphorylation sites'. Users can also specify whether or not to consider enzymes that can yield peptides containing some, but not all of the target sites.

**Figure 6:**
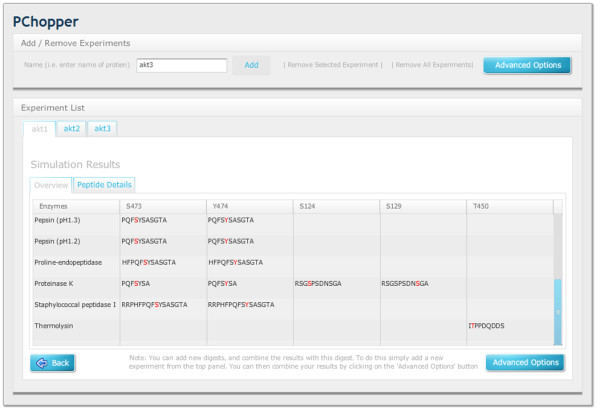
**Digest Results**. A simple results view that provides an overview of three simulated digests, namely an AKT1 digest, an AKT2 digest and an AKT3 digest. In this example details of the first digest are shown in a summary form. It outlines the enzymes that can be used to produce peptides containing the target sites that were selected in stage 2 of the workflow.

**Figure 7:**
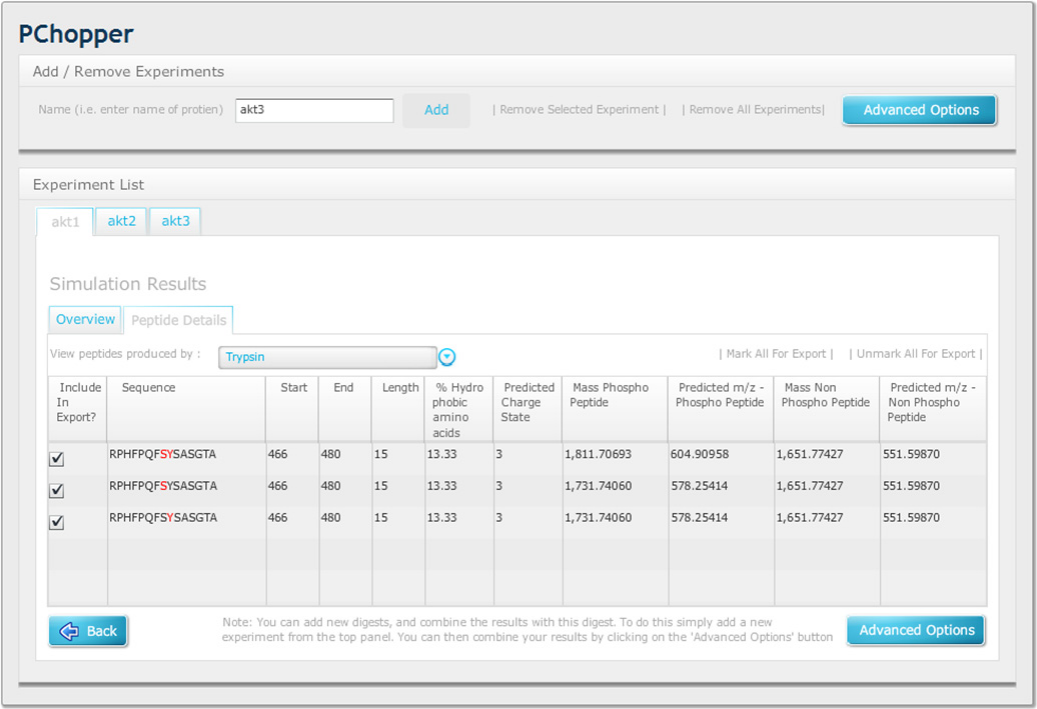
**Peptide Details**. A view showing the details of specific peptides. Users can select an enzyme and view the details of the peptides that were produced. For each peptide, the PChopper reports the length the peptide, the percentage hydrophobic amino acids, and the mass of the peptide (in both its phosphorylated and non phosphorylated forms).

**Figure 8:**
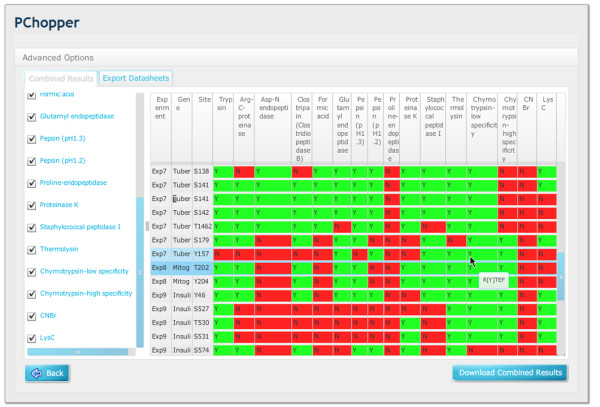
**Combined experiment results matrix**. A combined matrix showing the results from 9 experiments that target 52 sites. From this view it can be easily seen whether or not a particular enzyme can target a specific protein site. By placing the mouse-over a particular site the user can view the peptide sequence for any particular matrix entry.

**Figure 9:**
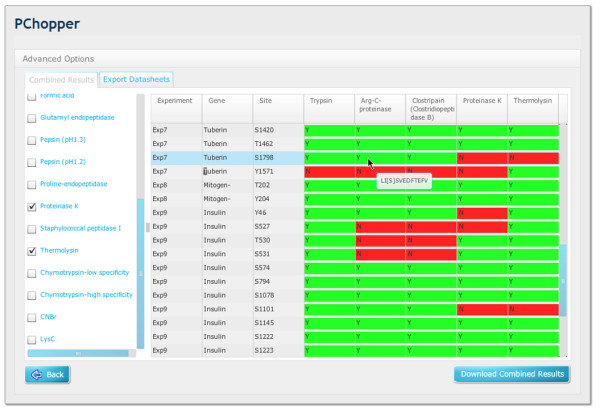
**Filtered combined experiment results matrix**. This figure includes the same output as Figure 8, but with a targeted set of enzymes that are selected to yield the best results.

PChopper provides a web based graphical interface, with an easy to follow workflow for running simulated digests. The workflow begins by specifying the name of the experiment. PChopper uses the term 'experiment' to describe the sequence that is to be digested, and the desired characteristics of the resultant peptides. For example, an experiment may involve a digest of AKT1, targeting phosphorylation sites at positions 473 and 308 so might be named 'AKT1 - S473, T308'. Once an experiment has been added, the user is prompted for a gene/protein name. This search term is then passed to the PhoshpoELM web-service as shown in Figure 2. The web-service then returns a list of matching entries, or an empty result if the search term could not be mapped to a gene/protein. For unsuccessful searches users are shown a popup stating that no search results could be found, and are prompted to search using a different term. For successful searches users are presented with a list of potential matches and are asked to select the correct entry based on the additional information that the search yielded. When the user has selected an entry, the amino acid sequence for the selected entry is displayed and the user can progress to the next step in the workflow (see Figure 3). The second step in the workflow involves asking the user to select the sites within the sequence they would like to target. This would typically be used for selecting regions within the sequence that are of interest, or sites within the sequence with post translational modifications that are of interest. Users have the option of selecting these manually and additionally PChopper can automatically identify known phosphorylation sites for human and mouse sequences. This automated process identifies all known phosphorylation sites, and the user can simply remove sites that are not of interest (see Figure 4). The third step in the workflow involves asking the user to specify any additional characteristics of the resultant peptides (length, exclusion criterion) and additional digest parameters. Users can adjust these based on their own requirements, or they can simply select the default settings and run the digest (see Figure 5). Once a digest has been performed, users are presented with the results in a matrix format (see Figure 6). Detailed information on each of the resultant peptides is also available on the peptide details tab (see Figure 7). This workflow can then be repeated for multiple proteins, and the results can be combined from the 'Advanced Options' screen. (see Figure 8 and 9).

### Result Formats

Once a simulated digest has been run, users are presented with an enzyme versus target site matrix. Each entry within the matrix shows the peptide that was produced by an enzyme for a specific target site. Additional details are also available for each of the resultant peptides. These include:

1. The starting position of the peptide within the sequence

2. The end position of the peptide within the sequence

3. The length of the peptide

4. The predicted charge state

5. The % of hydrophobic amino acids

6. The mass of the phospho-peptide

7. The mass of the non phospho-peptide

8. The predicted m/z ratio of the phospho-peptide

9. The predicted m/z ratio of the non phospho-peptide

10. The predicted retention time of the peptide (via the API)

In situations where users would like to monitor multiple sites on multiple proteins, it is useful to know the enzyme (or combination of enzymes) that are required to produce peptides with the required characteristics. In large studies this is especially true. PChopper's advanced results combination engine allows results from multiple digests to be combined into a single detailed summary view. From this view users can quickly identify the enzymes that can or cannot be used to target specific sites of interest. Users can then manually select/deselect enzymes, and export the combined results in csv (spreadsheet compatible) format. Additionally PChopper can automatically identify the most appropriate combination of enzymes and present this to the user in the form of a summarised datasheet. An additional datasheet is available as an export option, which provides full details on the digest, the protein/sequence that was digested, the enzymes that yielded peptides and the details of each of the peptides produced.

### Implementations Details

PChopper was developed as a Java application consisting of three distinct modules. Module 1 is responsible for running simulated digests and has no external dependencies other than the Java runtime environment. This has the advantage of cleanly separating the core business logic from any presentation or interaction logic. To run simulated digests, the module requires a protein sequence and a set of parameters describing the characteristics of the final peptide sequences. The system then 'digests' the sequence using the system's supported enzymes. The combination of a protein sequence and its digest parameters is called an 'experiment' and PChopper has the capability of running multiple experiments to identify suitable enzymes for use in monitoring multiple sites in multiple proteins.

**Figure 10:**
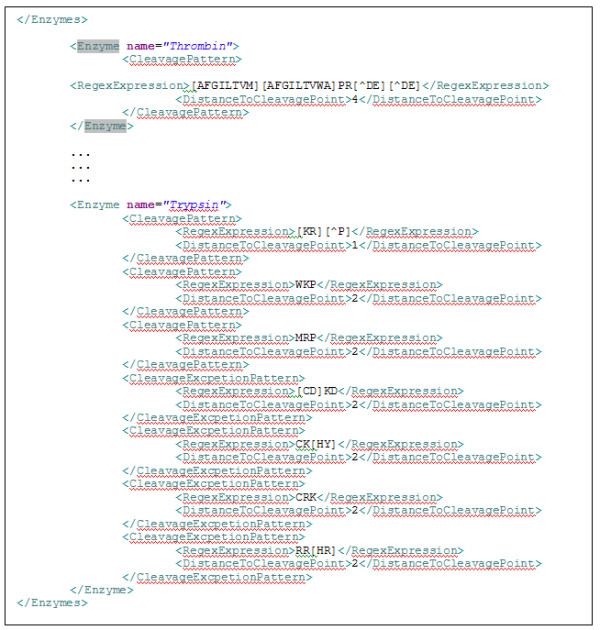
**Enzyme digest patterns**. An example of how cleavage patterns are defined by PChopper.

PChopper makes use of PeptideCutter's digest predictions, and stores them in a redefined XML format. PeptideCutter [2] is a web based tool from the ExPASy Proteomics Server that can predict potential cleavage sites caused by proteases and chemicals. When running a simulated digest, known digest cleavage patterns for 34 supported enzymes as defined by PeptideCutter are loaded from an XML file. The XML file stores the patterns as regular expressions as shown in Figure 10. Defining the patterns in this manner allows for separation of the patterns from the pattern processing engine, making the patterns easier to update and extend with new patterns as and when they become available. The patterns are applied by running a regular expression match of each cleavage pattern against the sequence being processed to identify the start of a pattern match. To determine the actual location of a cleavage site, the *DistanceToCleavagePoint *is added to the start position of the regular expression match index i.e. for the regular expression WKP, a distance of zero would define the cleavage as occurring before the W, a distance of 1 would define it as occurring between W and K, and so on. Once the cleavage sites are known, the peptides are defined as the amino acid sequences occurring between any two consecutive sets of identified cleavage sites, or between the first/last cleavage site and the beginning/end of the protein sequence. These peptides are then filtered based on the criterion specified by the user and presented as the output of the core module. Examples of filter criterion available in PChopper are presented in Table 1. The reasoning behind these filter criterion are described in [9].

**Table 1:** Available filters and parameters for simulated digests

Type of Filter/parameter	Description
Length filter	Filters out peptides outside of a defined range. i.e. peptides whose length is less than Len_min _or greater than Len_max _should be filtered from the final results. This can be customized to match the requirements of a particular experiment.

Problematic residue filter	Filters out peptides that contain residues that may be problematic. i.e. peptides that contain sulphur such (methionine and cysteine). Again this can be customized to match the requirements of a particular experiment.

Full dataset filter	Only lists results if the specified enzyme (or enzymes) is able to produce peptides that contain all of the specified residues.

Enzyme Multiplicity Parameter	Whether the simulated digest should use a single enzyme per run, or a combination of two enzymes for each run.

Phosphorylation Aware Cleaving Parameter	If this value is true, cleavages that are next to phosphorylation sites are not cleaved in the simulation.

Pair-wise Digest Parameter	Specifies if a pair-wise combination of enzymes should be used for each digest.

The second module has been developed as a search library whose primary role is to provide protein sequences and corresponding phosphorylation sites as parameters to Module 1. In keeping with the SOA theme, this module makes use of an existing search service, and wraps several of the methods behind an internal façade and makes them available via a simple Java interface. The service is provided by Phospho.Elm [10], which is a publicly available database of experimentally verified phosphorylation sites. It was chosen due to its wide usage [11,12], acclaimed accuracy [13-15] and because it exposes a web service [16]. It is also worth noting that Phospho.Elm is commonly used as a baseline for testing other phosphorylation prediction methods [14,11,17]. Figure 2 illustrates the information flow associated with this part of the system.

The third module has been developed as an interaction module to hide the complexities of interfacing Module 1 with the Module 2. This module has been designed in two parts, one focussing on human interactions and the other focussing on machine/programmatic interactions. For programmatic interactions a REST-based application end point was developed [18,19] which interfaces and wraps the methods available from modules one and two, allowing them to be invoked via simple http requests. For example, a GET request to the URL protein/akt1/digest results in the system invoking a simulated digest for AKT1, with the results being returned as an XML report. Details of the additional advantages of REST-based architectures are described in [8,19,20]. For a full list of available REST methods provided by PChopper, see Tables 1, 2, 3 and 4. For human interactions, a Flex based application endpoint was developed to provide a simple and intuitive system interface. The Flex GUI endpoint allows for a rich web-based solution that eliminates the need for client side installations and dependencies on natively installed software libraries. Since Flex compiles to Flash, it ensures the highest possible accessibility when compared to other rich browser-based plugins. The use of Flash as a runtime environment also eliminates the traditional problems associated with developing a web based system, such as having to account for differences in how browsers interpret and execute HTML and JavaScript functions. However, Flash inhibits the use of PChopper on some tablet PCs as there is currently limited support for Flash. Another limitation of Flash is that it cannot be easily indexed by search engines such as Google. While deep linking can be utilised to allow Flash content to be indexed, it is not a concern for PChopper as the applications 'states' do not require indexing..

**Table 2:** REST: Obtaining protein information

Description	Gets protein information that may be useful in distinguishing between similarly named biological entities. It is essentially a wrapper for the Phospho.ELM web service method.
HTTP-Method	GET

URL-Pattern	Application- Root >/protein/{protein}

Example URL	http://pchopper.lifesci.dundee.ac.uk/TmrcPortal/rest/properChopper/protein/akt

Example Results	< Substrates > < Substrate > < goTerms > nucleus</goTerms > < interaction > 1433Z_HUMAN</interaction > < interaction > TRAF2_HUMAN</interaction > < interaction > 1433T_HUMAN</interaction > < interaction > AKT1_HUMAN</interaction > < interaction > PDPK1_HUMAN</interaction > < interaction > RHEB_HUMAN</interaction > < interaction > Q56VH8_HUMAN</interaction > < interaction > 4EBP1_MOUSE</interaction > < interaction > FRAP_HUMAN</interaction > < interaction > EIF3F_MOUSE</interaction > < name > mTOR</name > </Substrate > < Substrate > < interaction > 1433S_HUMAN</interaction > < name > Rapamycin insensitive companion of mTOR </name > </Substrate > </Substrates >

**Table 3:** REST: Obtaining a protein sequence

Description	Gets the sequence associated with the specified protein. It is essentially a wrapper for the Phospho.ELM web service method. In situations where there is more than one match for the specified protein, the first match is used.
HTTP-Method	GET

URL-Pattern	Application- Root > /protein/{protein}/sequence

Example URL	http://pchopper.lifesci.dundee.ac.uk/TmrcPortal/rest/properChopper/protein/akt/sequence

Example Results	< Sequence > < description > Cell cycle control protein </description > < name > mTOR</name > < sequence > ...actual sequence ... </sequence > </Sequence >

**Table 4:** REST: Running a simulated digest

Description	Gets the results associated with a simulated digest
HTTP-Method	GET

URL-Pattern	Application- Root > /protein/{protein}/digest

Example URL	http://pchopper.lifesci.dundee.ac.uk/TmrcPortal/rest/properChopper/protein/akt/digest

URL-Paramters	peptideLenMin *- The minimum length of peptides to include in the results* peptideLenMax *- The maximum length of peptides to include in the results* peptideMustHaveAllResidues *- A boolean value that determines if the peptides produced from the digests should only be included if they are present within a single peptide* ignoreCleavAtPhosSite *- A boolean value that determines whether or not to ignore cleavages that occur next to a phosphorylated amino acid* usePairWiseEnzymeDigest *- A boolean value that determines whether or not to run run the simulated digest using combinations of two enzymes at a time* keyResidues *- The key amino acids in the sequence (that should be present in peptides)* exclusionChars *- The locations of amino acids that can prove to be problematic, peptides including these amino acids are filtered out*

Example URL	< Application- Root > /protein/akt/digest

Example Results	< DigestResults > < digest > < enzyme > Proteinase K</enzyme > < peptide > < indexEnd > 125</indexEnd > < sequenceHighlighted > RMNC [S] PT [S] QI</sequenceHighlighted > < molecularWeight > 1295.44408</molecularWeight > < percentageSolubility > 20.0</percentageSolubility > < retentionTime > 49.6</retentionTime > < sequence > RMNCSPTSQI</sequence > < indexStart > 116</indexStart > </peptide > - < peptide > < indexEnd > 306</indexEnd > < sequenceHighlighted > MK [T] F</sequenceHighlighted > < molecularWeight > 605.22843</molecularWeight > < percentageSolubility > 50.0</percentageSolubility > < retentionTime > 42.96</retentionTime > < sequence > MKTF</sequence > < indexStart > 303</indexStart > </peptide > ... other peptides ... </digest > ... other digests ... < sequence > ----Actual-Sequence---- </sequence > </DigestResults >

## Results

To demonstrate the capabilities of PChopper, we provide an example where monitoring of 52 phosphorylation sites in nine proteins (AKT1, AKT2, AKT3, GSK3α, GSK3β, FOXO1, TSC2, MAPK3, IRS1) is required. This would be a typical study where the phosphorylation sites of multiple enzymes in a signalling pathway need to be analysed in parallel and where we believe existing software would struggle to provide a simple solution. The proteins were analysed using experiments with the following parameters:

• No 'M' or 'C' in final peptides

• Peptide length between 5 and 30

• Ignore cleavages next to phosphorylation sites: True

• Only include results with all sites: False

The results of these nine experiments were presented to the user in the web-based viewer, and it allowed them to quickly and easily view the results from the nine experiments, and also to combine the results from the nine individual experiments in a single unified summary view. Additionally users can selectively export datasheets for additional information on each of the simulated experiments. Features of the single/combined results and the datasheets are outlined below.

### Single Digest Results

The results for any particular digest are presented immediately after a digest is completed. The results screen shows a list of enzymes, and the peptides that can be produced for each of the target sites. By scanning along a particular row in this table, it is very easy to identify the enzyme (or combination of enzymes) that are required to produce peptides for each of the required target sites (see Figure 6). A tab with further peptide details allows users to view the properties of each of the predicted peptides (see Figure 7).

### Combined Digest Results

PChopper can combine the results from multiple experiments into a single unified view. This view lists all proteins and their associated target sites, and maps these against the list of enzymes that were used to produce a selection matrix (see Figure 8). This matrix uses colour coding to help easily identify enzymes that can (or cannot) be used to produce a peptide containing a particular target site. A green box labelled 'Y' is used to indicate that an enzyme was able to produce a peptide which included the target site, and a red box labelled 'N' is used to indicate that the enzyme was not able to produce a peptide with the target site. Users can then select and de-select enzymes and export these as a CSV report. The CSV report reconfigures the data to group the results by enzyme, making it easier to see the enzymes that can be used to target specific sites of interest. Figure 8 shows the complete matrix, Figure 9 shows the cut down matrix.

### Datasheets

The details of each experiment can be downloaded as a datasheet. The datasheet contains additional information not included in the summary CSV file. For each simulated experiment the datasheet contains the following metadata used for the simulated digest:

• The name of the experiment

• The search term that was used to find the protein sequence

• The name of the matched protein that was used to retrieve the sequence

• The fragment filter criterion

• The peptide length criterion

• The sequence of the target protein, with the phosphorylation sites highlighted

• A list of all enzymes that yielded peptides that had the required characteristics.

The datasheets can be downloaded as a PDF report, and saved for future reference. Additional file 1 and additional file 2 are the datasheets associated with this series of experiments.

### Retention time calculations

Some scientists utilise retention time predictions in the prediction of SRM candidates. A challenge is that while tools are available to predict retention times for tryptic peptides, we are not aware of a tool which robustly predicts retention time for peptides including post-translational modifications, a key focus of PChopper.

At this point we have not implemented a retention time prediction algorithm in the GUI of PChopper, but we have made available the method published by Palmblad et al though the API [21]. Retention time prediction is generated as a property of each predicted peptide (see table 4). It should be noted that this method makes assumptions about the experimental conditions which may not be universally applicable.

## Conclusions

PChopper was developed to assist with designing studies for SRM-based protein phosphorylation analysis. While it includes features that are specific to phosphorylation, it is not constrained solely to digests involving this post-translational modification. PChopper can be used to target other post-translational modifications (that the user would have to enter manually) or simply to target regions within a protein sequence that are of interest. This can be done using a single enzyme, or with combinations of multiple enzymes. It was implemented using SOA architecture to produce a tool that is capable of quickly and easily predicting suitable enzymes and resulting peptides for SRM experiments. While there are other systems available such as MRMaid, PeptideCutter, SkyLine, ATAQS PChopper is unique from these. MRMaid does not include support for phosphopeptides as it actively filters out peptides with mass-altering post-transcriptional modifications. PeptideCutter can predict cleavage sites for enzymatic digests, but it lacks the ability to highlight peptides with phosphorylated amino acids. Skyline provides a complete end to end design workflow for SRM, but it is implemented using Microsoft's .Net client framework, making it inaccessible to platforms that cannot run .Net client applications, in comparison PChopper is fully web based. Similarly ATAQS does provide a complete end to end design workflow and additionally provides an application programming interface, however it is non-declarative and is bound to the implementation technologies; in comparison PChopper's programmatic access is declarative and is programming language agnostic.

## Availability and requirements

• **Project name: **PChopper

• **Project home page: **http://pchopper.lifesci.dundee.ac.uk

• **Operating system(s): **Platform independent

• **Programming language: **Java, Flex

• **Other requirements: **Web Browser with Flash player 10

• **License: **GPL

• **Any restrictions to use by non-academics: **None

## Authors' contributions

VA was the developer for the application. DC was the project manager for the system. JH and AA were involved in the requirements for the biological aspect of the system specification. All authors contributed to the final manuscript.

## Supplementary Material

Additional file 1 **Combined results from 9 experiments that target 52 sites**. Click here for file

Additional file 2 **The datasheet for the 9 experiments**. Click here for file

## References

[B1] Mead Ja Bianco L Ottone V Barton C Kay RG Lilley KS Bond NJ Bessant C MRMaid, the web-based tool for designing multiple reaction monitoring (MRM) transitions Molecular & cellular proteomics: MCP 2009 8 696 705 10.1074/mcp.M800192-MCP200 19011259 PMC2667351

[B2] Walke JM The Proteomics Protocols Handbook 2005 Humana Press 571 607

[B3] MacLean B Tomazela DM Shulman N Chambers M Finney GL Frewen B Kern R Tabb DL Liebler DC MacCoss MJ Skyline: an open source document editor for creating and analyzing targeted proteomics experiments Bioinformatics (Oxford, England) 2010 26 966 8 10.1093/bioinformatics/btq054 PMC2844992 20147306

[B4] Brusniak M-YK Kwok S-T Christiansen M Campbell D Reiter L Picotti P Kusebauch U Ramos H Deutsch EW Chen J Moritz RL Abersold R ATAQS: A computational software tool for high throughput transition optimization and validation for selected reaction monitoring mass spectrometry BMC Bioinformatics 2011 12 78 10.1186/1471-2105-12-78 21414234 PMC3213215

[B5] Cham J Bianco L Bessant C Free computational resources for designing selected reaction monitoring transitions Proteomics 2010 10 1106 1126 10.1002/pmic.200900396 20077412

[B6] Papazoglou MP Georgakopoulos D Service -oriented computing Communications of the ACM 2003 46 24 28

[B7] OReilly T What is Web 2.0: Design patterns and business models for the next generation of software 2005 http://papers.ssrn.com

[B8] Schroth C Janner T Web 2.0 and SOA: Converging Concepts Enabling the Internet of Services IT Professional 2007 9 36 41

[B9] Anderson L Hunter CL Quantitative Mass Spectrometric Multiple Reaction Monitoring Assays for Major Plasma Proteins Mol Cell Proteomics 2006 5 573 88 16332733 10.1074/mcp.M500331-MCP200

[B10] Diella F Cameron S Gemünd C Linding R Via A Kuster B Sicheritz-Ponten T Blom N Ginson TJ Phospho.ELM: a database of experimentally verified phosphorylation sites in eukaryotic proteins BMC bioinformatics 2004 5 79 10.1186/1471-2105-5-79 15212693 PMC449700

[B11] Lee T-Y Huang H-D Hung J-H Huang H-Y Yang Y-S Wang T-H dbPTM: an information repository of protein post-translational modification Nucleic acids research 2006 34 D622 D627 10.1093/nar/gkj083 16381945 PMC1347446

[B12] Davey NE Edwards RJ Shields DC Estimation and efficient computation of the true probability of recurrence of short linear protein sequence motifs in unrelated proteins BMC bioinformatics 2010 11 14 10.1186/1471-2105-11-14 20055997 PMC2819990

[B13] Gould CM Diella F Via A Puntervoll P Gemünd C Chabanis-Davidson S Michael S Sayadi A Bryne JC Chica C Seiler M Davey NE Haslam N Weatheritt RJ Budd A Hughes T Pas J Rychlewski L Trave G Aasland R Helmer-Citterich M Linding R Gibson TJ ELM: the status of the 2010 eukaryotic linear motif resource Nucleic acids research 2010 38 D167 D180 10.1093/nar/gkp1016 19920119 PMC2808914

[B14] Dang TH Van Leemput K Verschoren A Laukens K Prediction of kinase-specific phosphorylation sites using conditional random fields Bioinformatics (Oxford, England) 2008 24 2857 64 10.1093/bioinformatics/btn546 PMC2639296 18940828

[B15] Xue Y Ren J Gao X Jin C Wen L Yao X GPS 2.0: Prediction of kinase-specific phosphorylation sites in hierarchy Mol Cell Proteomics 2008 7 1598 1608 10.1074/mcp.M700574-MCP200 18463090 PMC2528073

[B16] Diella F Gould CM Chica C Via A Gibson TJ Phospho.ELM: a database of phosphorylation sites - update 2008 Nucleic Acids Research 2008 36 D240 D244 17962309 10.1093/nar/gkm772 PMC2238828

[B17] Zhou FF Xue Y Chen GL Yao X GPS: a novel group-based phosphorylation predicting and scoring method Biochemical and Biophysical Research Communications 2004 325 1443 1448 10.1016/j.bbrc.2004.11.001 15555589

[B18] Battle R Benson E Bridging the semantic Web and Web 2.0 with Representational State Transfer (REST) Web Semantics: Science, Services and Agents on the World Wide Web 2008 6 61 69 10.1016/j.websem.2007.11.002

[B19] Fielding RT Taylor RN Principled design of the modern Web architecture ACM Transactions on Internet Technology (TOIT) 2002 2 115 150 10.1145/514183.514185

[B20] Goth G Critics Say Web Services Need a REST IEEE Distributed Systems Online 2004 5 1 1

[B21] Palmblad M Ramström M Markides KE Håkansson P Bergquist J Prediction of Chromatographic Retention and Protein Identification in Liquid Chromatography/Mass Spectrometry Analytical Chemistry 2002 74 5826 5830 10.1021/ac0256890 12463368

