# Supplementary material for: PChopper: high throughput peptide prediction for MRM/SRM transition design
Source: BMC Bioinformatics. 2011 Aug 15;12:338. doi: 10.1186/1471-2105-12-338 (PMC3230909; doi:10.1186/1471-2105-12-338)
Supplement: Additional file 2 — The datasheet for the 9 experiments. [file 1471-2105-12-338-S2.PDF]

PChopper

Experiment Name

Exp1

Search Term

Akt1

Protein Name

PKB-alpha

Fragment Filter Criterion

No M

No C

Fragment Length Criterion

Between 5 and 30

Target Protein Sequence

1	MSDVAIVKEGWLHKRGEYIKTWRPRYFLLKNDGTFIGYKERPQDVDQREA
51	PLNNFSVAQCQLMKTERPRPNTFIIRCLQWTTVIERTFHVETPEEREEWT
101	TAIQTVADGLKKQEEEEMDFRSG**[S124]**PSDN**[S129]**GAEEMEVSLAKPKHRVTMNEF
151	EYLKLLGKGTFGKVILVKEKATGRYYAMKILKKEVIVAKDEVAHTLTENR
201	VLQNSRHPFLTALKYSFQTHDRLCFVMEYANGGELFFHLSRERVFSEDRA
251	RFYGAEIVSALDYLHSEKNVVYRDLKLENLMLDKDGHIKITDFGLCKEGI
301	KDGATMK**[T308]**FCGTPEYLAPEVLEDNDYGRAVDWWGLGVVMYEMMCGRLPFY
351	NQDHEKLFELILMEEIRFPRTLGPEAKSLLSGLLKKDPKQRLGGGSEDAK
401	EIMQHRFFAGIVWQHVYEKKLSPPFKPQVTSETDTRYFDEEFTAQMITI**[T450]**
451	PPDQDDSMECVDSERRPHFPQF**[S473][Y474]**SASGTA

Trypsin							
				Phospho Peptide		Non Phospho Peptide	
Peptide Sequence	Length	% hydro - phobic amino acids	Predicted Charge State(z)	Mono Isotpoic mass	Predicted m/z	Theoretical Mass	Predicted m/z
RPHFPQF**[S473][Y474]**SASGTA	15	13.3	3.0	1811.7069	604.9096	1651.7743	551.5987
RPHFPQF**[S473]**YSASGTA	15	13.3	3.0	1731.7406	578.2541	1651.7743	551.5987
RPHFPQFS**[Y474]**SASGTA	15	13.3	3.0	1731.7406	578.2541	1651.7743	551.5987

Arg-C-proteinase							
				Phospho Peptide		Non Phospho Peptide	
Peptide Sequence	Length	% hydro - phobic amino acids	Predicted Charge State(z)	Mono Isotpoic mass	Predicted m/z	Theoretical Mass	Predicted m/z
PHFPQF**[S473][Y474]**SASGTA	14	14.3	2.0	1655.6058	828.8102	1495.6732	748.8439
PHFPQF**[S473]**YSASGTA	14	14.3	2.0	1575.6395	788.8270	1495.6732	748.8439
PHFPQFS**[Y474]**SASGTA	14	14.3	2.0	1575.6395	788.8270	1495.6732	748.8439

Asp-N endopeptidase							
				Phospho Peptide		Non Phospho Peptide	
Peptide Sequence	Length	% hydro - phobic amino acids	Predicted Charge State(z)	Mono Isotpoic mass	Predicted m/z	Theoretical Mass	Predicted m/z
DFRSG**[S124]**PS	8	12.5	2.0	931.3437	466.6791	851.3773	426.6959
DSERRPHFPQF**[S473][Y474]**SASGTA	19	10.5	4.0	2298.9096	575.7347	2138.9770	535.7515
DSERRPHFPQF**[S473]**YSASGTA	19	10.5	4.0	2218.9433	555.7431	2138.9770	535.7515
DSERRPHFPQFS**[Y474]**SASGTA	19	10.5	4.0	2218.9433	555.7431	2138.9770	535.7515

Clostripain (Clostridiopeptidase B)							
				Phospho Peptide		Non Phospho Peptide	
Peptide Sequence	Length	% hydro - phobic amino acids	Predicted Charge State(z)	Mono Isotpoic mass	Predicted m/z	Theoretical Mass	Predicted m/z
PHFPQF**[S473][Y474]**SASGTA	14	14.3	2.0	1655.6058	828.8102	1495.6732	748.8439
PHFPQF**[S473]**YSASGTA	14	14.3	2.0	1575.6395	788.8270	1495.6732	748.8439
PHFPQFS**[Y474]**SASGTA	14	14.3	2.0	1575.6395	788.8270	1495.6732	748.8439

Formic acid							
				Phospho Peptide		Non Phospho Peptide	
Peptide Sequence	Length	% hydro - phobic amino acids	Predicted Charge State(z)	Mono Isotpoic mass	Predicted m/z	Theoretical Mass	Predicted m/z
FRSG**[S124]**PSD	8	12.5	2.0	931.3437	466.6791	851.3773	426.6959
SERRPHFPQF**[S473][Y474]**SASGTA	18	11.1	4.0	2183.8827	546.9779	2023.9500	506.9948
SERRPHFPQF**[S473]**YSASGTA	18	11.1	4.0	2103.9163	526.9864	2023.9500	506.9948
SERRPHFPQFS**[Y474]**SASGTA	18	11.1	4.0	2103.9163	526.9864	2023.9500	506.9948

Glutamyl endopeptidase							
				Phospho Peptide		Non Phospho Peptide	
Peptide Sequence	Length	% hydro - phobic amino acids	Predicted Charge State(z)	Mono Isotpoic mass	Predicted m/z	Theoretical Mass	Predicted m/z
RRPHFPQF**[S473][Y474]**SASGTA	16	12.5	4.0	1967.8080	492.9593	1807.8754	452.9761
RRPHFPQF**[S473]**YSASGTA	16	12.5	4.0	1887.8417	472.9677	1807.8754	452.9761
RRPHFPQFS**[Y474]**SASGTA	16	12.5	4.0	1887.8417	472.9677	1807.8754	452.9761

Pepsin (pH1.3)							
				Phospho Peptide		Non Phospho Peptide	
Peptide Sequence	Length	% hydro - phobic amino acids	Predicted Charge State(z)	Mono Isotpoic mass	Predicted m/z	Theoretical Mass	Predicted m/z
PQF**[S473][Y474]**SASGTA	11	9.1	1.0	1274.4257	1275.4330	1114.4931	1115.5004
PQF**[S473]**YSASGTA	11	9.1	1.0	1194.4594	1195.4667	1114.4931	1115.5004
PQFS**[Y474]**SASGTA	11	9.1	1.0	1194.4594	1195.4667	1114.4931	1115.5004

Pepsin (pH1.2)							
				Phospho Peptide		Non Phospho Peptide	
Peptide Sequence	Length	% hydro - phobic amino acids	Predicted Charge State(z)	Mono Isotpoic mass	Predicted m/z	Theoretical Mass	Predicted m/z
PQF**[S473][Y474]**SASGTA	11	9.1	1.0	1274.4257	1275.4330	1114.4931	1115.5004
PQF**[S473]**YSASGTA	11	9.1	1.0	1194.4594	1195.4667	1114.4931	1115.5004
PQFS**[Y474]**SASGTA	11	9.1	1.0	1194.4594	1195.4667	1114.4931	1115.5004

Proline-endopeptidase							
				Phospho Peptide		Non Phospho Peptide	
Peptide Sequence	Length	% hydro - phobic amino acids	Predicted Charge State(z)	Mono Isotpoic mass	Predicted m/z	Theoretical Mass	Predicted m/z
HFPQF**[S473][Y474]**SASGTA	13	15.4	2.0	1558.5531	780.2838	1398.6204	700.3175
HFPQF**[S473]**YSASGTA	13	15.4	2.0	1478.5867	740.3006	1398.6204	700.3175
HFPQFS**[Y474]**SASGTA	13	15.4	2.0	1478.5867	740.3006	1398.6204	700.3175

Proteinase K							
				Phospho Peptide		Non Phospho Peptide	
Peptide Sequence	Length	% hydro - phobic amino acids	Predicted Charge State(z)	Mono Isotpoic mass	Predicted m/z	Theoretical Mass	Predicted m/z
RSG**[S124]**PSDN**[S129]**GA	11	0.0	2.0	1193.3751	597.6948	1033.4424	517.7285
PQF**[S473][Y474]**SA	7	14.3	1.0	958.2875	959.2947	798.3548	799.3621
RSG**[S124]**PSDNSGA	11	0.0	2.0	1113.4088	557.7117	1033.4424	517.7285
RSGSPSDN**[S129]**GA	11	0.0	2.0	1113.4088	557.7117	1033.4424	517.7285
PQF**[S473]**YSA	7	14.3	1.0	878.3211	879.3284	798.3548	799.3621
PQFS**[Y474]**SA	7	14.3	1.0	878.3211	879.3284	798.3548	799.3621

Staphylococcal peptidase I							
				Phospho Peptide		Non Phospho Peptide	
Peptide Sequence	Length	% hydro - phobic amino acids	Predicted Charge State(z)	Mono Isotpoic mass	Predicted m/z	Theoretical Mass	Predicted m/z
RRPHFPQF**[S473][Y474]**SASGTA	16	12.5	4.0	1967.8080	492.9593	1807.8754	452.9761
RRPHFPQF**[S473]**YSASGTA	16	12.5	4.0	1887.8417	472.9677	1807.8754	452.9761
RRPHFPQFS**[Y474]**SASGTA	16	12.5	4.0	1887.8417	472.9677	1807.8754	452.9761

Thermolysin							
				Phospho Peptide		Non Phospho Peptide	
Peptide Sequence	Length	% hydro - phobic amino acids	Predicted Charge State(z)	Mono Isotpoic mass	Predicted m/z	Theoretical Mass	Predicted m/z
I**[T450]**PPDQDDS	9	11.1	1.0	1066.3856	1067.3929	986.4192	987.4265

PChopper

Experiment Name

Exp2

Search Term

Akt2

Protein Name

PKB-beta

Fragment Filter Criterion

No M

No C

Fragment Length Criterion

Between 5 and 30

Target Protein Sequence

1	MNEVSVIKEGWLHKRGEYIKTWRPRYFLLKSDGSFIGYKERPEAPDQTLP
51	PLNNFSVAECQLMKTERPRPNTFVIRCLQWTTVIERTFHVDSPDEREEWM
101	RAIQMVANSLKQRAPGEDPMDYKCG**[S126]**PSDSSTTEEMEVAVSKARAKVTMN
151	DFDYLKLLGKGTFGKVILVREKATGRYYAMKILRKEVIIAKDEVAHTVTE
201	SRVLQNTRHPFLTALKYAFQTHDRLCFVMEYANGGELFFHLSRERVFTEE
251	RARFYGAEIVSALEYLHSRDVVYRDIKLENLMLDKDGHIKITDFGLCKEG
301	ISDGATMK**[T309]**FCGTPEYLAPEVLEDNDYGRAVDWWGLGVVMYEMMCGRLPF
351	YNQDHERLFELILMEEIRFPRTLSPEAKSLLAGLLKKDPKQRLGGGPSDA
401	KEVMEHRFFLSINWQDVVQKKLLPPFKPQVTSEVDTRYFDDEFTAQSITI
451	**[T451]**PPDRYDSLGLLELDQRTHFPQF**[S474]**YSASIRE

Trypsin							
				Phospho Peptide		Non Phospho Peptide	
Peptide Sequence	Length	% hydro - phobic amino acids	Predicted Charge State(z)	Mono Isotpoic mass	Predicted m/z	Theoretical Mass	Predicted m/z
YFDDEFTAQSITI**[T451]**PPDR	18	22.2	2.0	2194.9460	1098.4803	2114.9796	1058.4971
THFPQF**[S474]**YSASIR	13	23.1	3.0	1619.7133	540.9117	1539.7470	514.2563

Chymotrypsin-low specificity							
				Phospho Peptide		Non Phospho Peptide	
Peptide Sequence	Length	% hydro - phobic amino acids	Predicted Charge State(z)	Mono Isotpoic mass	Predicted m/z	Theoretical Mass	Predicted m/z
TAQSITI**[T451]**PPDRY	13	15.4	2.0	1541.7127	771.8636	1461.7463	731.8804
DQRTHFPQF**[S474]**Y	11	18.2	3.0	1504.6136	502.5452	1424.6473	475.8897

Chymotrypsin-high specificity							
				Phospho Peptide		Non Phospho Peptide	
Peptide Sequence	Length	% hydro - phobic amino acids	Predicted Charge State(z)	Mono Isotpoic mass	Predicted m/z	Theoretical Mass	Predicted m/z
TAQSITI**[T451]**PPDRY	13	15.4	2.0	1541.7127	771.8636	1461.7463	731.8804
DSLGLLELDQRTHFPQF**[S474]**Y	19	31.6	3.0	2345.0729	782.6982	2265.1066	756.0428

Arg-C-proteinase							
				Phospho Peptide		Non Phospho Peptide	
Peptide Sequence	Length	% hydro - phobic amino acids	Predicted Charge State(z)	Mono Isotpoic mass	Predicted m/z	Theoretical Mass	Predicted m/z
YFDDEFTAQSITI**[T451]**PPDR	18	22.2	2.0	2194.9460	1098.4803	2114.9796	1058.4971
THFPQF**[S474]**YSASIR	13	23.1	3.0	1619.7133	540.9117	1539.7470	514.2563

Asp-N endopeptidase							
				Phospho Peptide		Non Phospho Peptide	
Peptide Sequence	Length	% hydro - phobic amino acids	Predicted Charge State(z)	Mono Isotpoic mass	Predicted m/z	Theoretical Mass	Predicted m/z
DEFTAQSITI**[T451]**PP	13	23.1	1.0	1498.6592	1499.6665	1418.6929	1419.7002
DQRTHFPQF**[S474]**YSASIRE	17	17.6	4.0	2147.9426	537.9929	2067.9762	518.0013

Clostripain (Clostridiopeptidase B)							
				Phospho Peptide		Non Phospho Peptide	
Peptide Sequence	Length	% hydro - phobic amino acids	Predicted Charge State(z)	Mono Isotpoic mass	Predicted m/z	Theoretical Mass	Predicted m/z
YFDDEFTAQSITI**[T451]**PPDR	18	22.2	2.0	2194.9460	1098.4803	2114.9796	1058.4971
THFPQF**[S474]**YSASIR	13	23.1	3.0	1619.7133	540.9117	1539.7470	514.2563

Formic acid							
				Phospho Peptide		Non Phospho Peptide	
Peptide Sequence	Length	% hydro - phobic amino acids	Predicted Charge State(z)	Mono Isotpoic mass	Predicted m/z	Theoretical Mass	Predicted m/z
EFTAQSITI**[T451]**PPD	13	23.1	1.0	1498.6592	1499.6665	1418.6929	1419.7002
QRTHFPQF**[S474]**YSASIRE	16	18.8	4.0	2032.9156	509.2362	1952.9493	489.2446

Glutamyl endopeptidase							
				Phospho Peptide		Non Phospho Peptide	
Peptide Sequence	Length	% hydro - phobic amino acids	Predicted Charge State(z)	Mono Isotpoic mass	Predicted m/z	Theoretical Mass	Predicted m/z
FTAQSITI**[T451]**PPDRYDSLGLLE	21	28.6	2.0	2416.1563	1209.0854	2336.1900	1169.1023
LDQRTHFPQF**[S474]**YSASIRE	18	22.2	4.0	2261.0266	566.2639	2181.0603	546.2723

Pepsin (pH1.3)							
				Phospho Peptide		Non Phospho Peptide	
Peptide Sequence	Length	% hydro - phobic amino acids	Predicted Charge State(z)	Mono Isotpoic mass	Predicted m/z	Theoretical Mass	Predicted m/z
TAQSITI**[T451]**PPDRY	13	15.4	2.0	1541.7127	771.8636	1461.7463	731.8804
PQF**[S474]**Y	5	20.0	1.0	720.2520	721.2593	640.2857	641.2929

Pepsin (pH1.2)							
				Phospho Peptide		Non Phospho Peptide	
Peptide Sequence	Length	% hydro - phobic amino acids	Predicted Charge State(z)	Mono Isotpoic mass	Predicted m/z	Theoretical Mass	Predicted m/z
TAQSITI**[T451]**PPDRYDS	15	13.3	2.0	1743.7716	872.8931	1663.8053	832.9099
PQF**[S474]**YSASIRE	11	18.2	2.0	1363.5809	682.7977	1283.6146	642.8146

Proteinase K							
				Phospho Peptide		Non Phospho Peptide	
Peptide Sequence	Length	% hydro - phobic amino acids	Predicted Charge State(z)	Mono Isotpoic mass	Predicted m/z	Theoretical Mass	Predicted m/z
I**[T451]**PPDRY	7	14.3	2.0	940.4055	471.2100	860.4392	431.2269
PQF**[S474]**Y	5	20.0	1.0	720.2520	721.2593	640.2857	641.2929

Staphylococcal peptidase I							
				Phospho Peptide		Non Phospho Peptide	
Peptide Sequence	Length	% hydro - phobic amino acids	Predicted Charge State(z)	Mono Isotpoic mass	Predicted m/z	Theoretical Mass	Predicted m/z
FTAQSITI**[T451]**PPDRYDSLGLLE	21	28.6	2.0	2416.1563	1209.0854	2336.1900	1169.1023
LDQRTHFPQF**[S474]**YSASIRE	18	22.2	4.0	2261.0266	566.2639	2181.0603	546.2723

Thermolysin							
				Phospho Peptide		Non Phospho Peptide	
Peptide Sequence	Length	% hydro - phobic amino acids	Predicted Charge State(z)	Mono Isotpoic mass	Predicted m/z	Theoretical Mass	Predicted m/z
I**[T451]**PPDRYDS	9	11.1	2.0	1142.4645	572.2395	1062.4982	532.2564

*P*Chopper

Experiment Name

Exp3

Search Term

Akt3

Protein Name

PKB-gamma

Fragment Filter Criterion

No M

No C

Fragment Length Criterion

Between 5 and 30

Target Protein Sequence

```
1       MSDVTIVKEGWVQKRGEYIKNWRPRYFLLKTDGSFIGYKEKPQDVDLPYP
51      LNNFSVAKCQLMKTERPKPNTFIIRCLQWTTVIERTFHVDTPEEREEWTE
101     AIQAVADRLQRQEEERMNC[S120]PTSQIDNIGEEEMDASTTHHKRKTMNDFDY
151     LKLLGKGTFGKVILVREKASGKYYAMKILKKEVIIAKDEVAHTLTESRVL
201     KNTRHPFLTSLKYSFQTKDRLCFVMEYVNGGELFFHLSRERVFSEDRTRF
251     YGAEIVSALDYLHSGKIVYRDLKLENLMLDKDGHIKITDFGLCKEGITDA
301     ATMK[T305]FCGTPEYLAPEVLEDNDYGRAVDWWGLGVVMYEMMCGRLPFYNQD
351     HEKLFELILMEDIKFPRTLSSDAKSLLSGLLIKDPNKRLGGGPDDAKEIM
401     RHSFFSGVNWQDVYDKKLVPPFKPQVTSETDTRYFDEEFTAQTITI[T447]PPE
451     KYDEDGMDCMDNERRPHFPQF[S472]YSASGRE
```

Trypsin							
				Phospho Peptide		Non Phospho Peptide	
Peptide Sequence	Length	% hydro - phobic amino acids	Predicted Charge State(z)	Mono Isotpoic mass	Predicted m/z	Theoretical Mass	Predicted m/z
YFDEEFTAQTITI**[T447]**PPEK	18	22.2	2.0	2208.9868	1105.5007	2129.0204	1065.5175
RPHFPQF**[S472]**YSASGR	14	14.3	4.0	1715.7569	429.9465	1635.7906	409.9549

Chymotrypsin-low specificity							
				Phospho Peptide		Non Phospho Peptide	
Peptide Sequence	Length	% hydro - phobic amino acids	Predicted Charge State(z)	Mono Isotpoic mass	Predicted m/z	Theoretical Mass	Predicted m/z
TAQTITI**[T447]**PPEKY	13	15.4	2.0	1541.7378	771.8762	1461.7715	731.8930
DNERRPHFPQF**[S472]**Y	13	15.4	4.0	1771.7468	443.9440	1691.7804	423.9524

Chymotrypsin-high specificity							
				Phospho Peptide		Non Phospho Peptide	
Peptide Sequence	Length	% hydro - phobic amino acids	Predicted Charge State(z)	Mono Isotpoic mass	Predicted m/z	Theoretical Mass	Predicted m/z
TAQTITI**[T447]**PPEKY	13	15.4	2.0	1541.7378	771.8762	1461.7715	731.8930

Arg-C-proteinase							
				Phospho Peptide		Non Phospho Peptide	
Peptide Sequence	Length	% hydro - phobic amino acids	Predicted Charge State(z)	Mono Isotpoic mass	Predicted m/z	Theoretical Mass	Predicted m/z
PHFPQF**[S472]**YSASGR	13	15.4	3.0	1559.6558	520.8925	1479.6895	494.2371

Asp-N endopeptidase							
				Phospho Peptide		Non Phospho Peptide	
Peptide Sequence	Length	% hydro - phobic amino acids	Predicted Charge State(z)	Mono Isotpoic mass	Predicted m/z	Theoretical Mass	Predicted m/z
DEEFTAQTITI**[T447]**PPEKY	17	17.6	2.0	2061.9183	1031.9665	1981.9520	991.9833
DNERRPHFPQF**[S472]**YSASGRE	19	10.5	5.0	2359.0131	472.8099	2279.0468	456.8166

Clostripain (Clostridiopeptidase B)							
				Phospho Peptide		Non Phospho Peptide	
Peptide Sequence	Length	% hydro - phobic amino acids	Predicted Charge State(z)	Mono Isotpoic mass	Predicted m/z	Theoretical Mass	Predicted m/z
PHFPQF**[S472]**YSASGR	13	15.4	3.0	1559.6558	520.8925	1479.6895	494.2371

CNBr							
				Phospho Peptide		Non Phospho Peptide	
Peptide Sequence	Length	% hydro - phobic amino acids	Predicted Charge State(z)	Mono Isotpoic mass	Predicted m/z	Theoretical Mass	Predicted m/z
DNERRPHFPQF**[S472]**YSASGRE	19	10.5	5.0	2359.0131	472.8099	2279.0468	456.8166

Formic acid							
				Phospho Peptide		Non Phospho Peptide	
Peptide Sequence	Length	% hydro - phobic amino acids	Predicted Charge State(z)	Mono Isotpoic mass	Predicted m/z	Theoretical Mass	Predicted m/z
EEFTAQTITI**[T447]**PPEKYD	17	17.6	2.0	2061.9184	1031.9665	1981.9520	991.9833
NERRPHFPQF**[S472]**YSASGRE	18	11.1	5.0	2243.9862	449.8045	2164.0198	433.8112

Glutamyl endopeptidase							
				Phospho Peptide		Non Phospho Peptide	
Peptide Sequence	Length	% hydro - phobic amino acids	Predicted Charge State(z)	Mono Isotpoic mass	Predicted m/z	Theoretical Mass	Predicted m/z
FTAQTITI**[T447]**PPE	12	25.0	1.0	1397.6479	1398.6552	1317.6816	1318.6889
RRPHFPQF**[S472]**YSASGRE	16	12.5	5.0	2000.9006	401.1874	1920.9343	385.1941

LysC							
				Phospho Peptide		Non Phospho Peptide	
Peptide Sequence	Length	% hydro - phobic amino acids	Predicted Charge State(z)	Mono Isotpoic mass	Predicted m/z	Theoretical Mass	Predicted m/z
PQVTSETDTRYFDEEFTAQTITI**[T447]**PPEK	28	17.9	3.0	3323.5122	1108.8447	3243.5459	1082.1892

Pepsin (pH1.3)							
				Phospho Peptide		Non Phospho Peptide	
Peptide Sequence	Length	% hydro - phobic amino acids	Predicted Charge State(z)	Mono Isotpoic mass	Predicted m/z	Theoretical Mass	Predicted m/z
TAQTITI**[T447]**PPEK	12	16.7	2.0	1378.6745	690.3445	1298.7082	650.3614
PQF**[S472]**Y	5	20.0	1.0	720.2520	721.2593	640.2857	641.2929

Pepsin (pH1.2)							
				Phospho Peptide		Non Phospho Peptide	
Peptide Sequence	Length	% hydro - phobic amino acids	Predicted Charge State(z)	Mono Isotpoic mass	Predicted m/z	Theoretical Mass	Predicted m/z
PQF**[S472]**YSASGRE	11	9.1	2.0	1307.5183	654.7664	1227.5520	614.7833

Proline-endopeptidase							
				Phospho Peptide		Non Phospho Peptide	
Peptide Sequence	Length	% hydro - phobic amino acids	Predicted Charge State(z)	Mono Isotpoic mass	Predicted m/z	Theoretical Mass	Predicted m/z
HFPQF**[S472]**YSASGRE	13	15.4	3.0	1591.6456	531.5558	1511.6793	504.9004

Proteinase K							
				Phospho Peptide		Non Phospho Peptide	
Peptide Sequence	Length	% hydro - phobic amino acids	Predicted Charge State(z)	Mono Isotpoic mass	Predicted m/z	Theoretical Mass	Predicted m/z
I**[T447]**PPE	5	20.0	1.0	635.2567	636.2640	555.2904	556.2977
PQF**[S472]**Y	5	20.0	1.0	720.2520	721.2593	640.2857	641.2929

Staphylococcal peptidase I							
				Phospho Peptide		Non Phospho Peptide	
Peptide Sequence	Length	% hydro - phobic amino acids	Predicted Charge State(z)	Mono Isotpoic mass	Predicted m/z	Theoretical Mass	Predicted m/z
EFTAQTITI**[T447]**PPE	13	23.1	1.0	1526.6905	1527.6978	1446.7242	1447.7315
RRPHFPQF**[S472]**YSASGRE	16	12.5	5.0	2000.9006	401.1874	1920.9343	385.1941

Thermolysin							
				Phospho Peptide		Non Phospho Peptide	
Peptide Sequence	Length	% hydro - phobic amino acids	Predicted Charge State(z)	Mono Isotpoic mass	Predicted m/z	Theoretical Mass	Predicted m/z
I**[T447]**PPEKYDEDG	11	9.1	2.0	1342.5330	672.2738	1262.5666	632.2906

PChopper

Experiment Name

Exp4

Search Term

Gsk3a

Protein Name

GSK-3 alpha

Fragment Filter Criterion

No M

No C

Fragment Length Criterion

Between 5 and 30

Target Protein Sequence

```
1       MSGGGPSGGGPGGSGRARTS[S21]FAEPGGGGGGGGGGPGGSASGPGGTGGGK
51      ASVGAMGGGVGASSSGGGPSGSGGGGSGGPGAGTSFPPPGVKLGRDSGKV
101     TTVVATLGQGPERSQEVAYTDIKVIGNGSFGVVYQARLAETRELVAIKKV
151     LQDKRFKNRELQIMRKLDHCNIVRLRYFFYSSGEKKDELYLNLVLEYVPE
201     TVYRVARHFTKAKLIIPIIYVKVYMYQLFRSLAYIHSQGVCHRDIKPQNL
251     LVDPDTAVLKLCDFGSAKQLVRGEPNVS[Y279]ICSRYYRAPELIFGATDYTSS
301     IDVWSAGCVLAELLLGQPIFPGDSGVDQLVEIIKVLGTPTREQIREMNPN
351     YTEFKFPQIKAHPWTKVFKSRTPPEAIALCSSLLEYTPSSRLSPLEACAH
401     SFFDELRSLGTQLPNNRPLPPLFNFSPGELSIQPSLNAILIPPHLRSPSG
451     PATLTSSSQALTETQTGQDWQAPDATPTLTNSS
```

Chymotrypsin-low specificity							
				Phospho Peptide		Non Phospho Peptide	
Peptide Sequence	Length	% hydro - phobic amino acids	Predicted Charge State(z)	Mono Isotpoic mass	Predicted m/z	Theoretical Mass	Predicted m/z
SGGGPSGGGPGGSGRARTS**[S21]**F	21	4.8	3.0	1871.7911	624.9376	1791.8248	598.2822

Thermolysin							
				Phospho Peptide		Non Phospho Peptide	
Peptide Sequence	Length	% hydro - phobic amino acids	Predicted Charge State(z)	Mono Isotpoic mass	Predicted m/z	Theoretical Mass	Predicted m/z
ARTS**[S21]**FAEPGGGGGGGGGPGGS	23	4.4	2.0	1912.7700	957.3923	1832.8037	917.4091

*P*Chopper

Experiment Name

Exp5

Search Term

Gsk3b

Protein Name

GSK-3 beta

Fragment Filter Criterion

No M

No C

Fragment Length Criterion

Between 5 and 30

Target Protein Sequence

1	MSGRPRTT**[S9]**FAESCKPVQQP**[S21]**AFGSMKVSRDKDGSKVTTVVA**[T43]**PGQGPDR
51	PQEVSYTDTKVIGNGSFGVVYQAKLCDSGELVAIKKVLQDKRFKNRELQI
101	MRKLDHCNIVRLRYFFYSSGEKKDEVYLNLVLDYVPETVYRVARHYSRAK
151	QTLPVIYVKLYMYQLFRSLAYIHSFGICHRDIKPQNLLLDPDTAVLKLCD
201	FGSAKQLVRGEPNVS**[Y216]**ICSRYYRAPELIFGATDYTSSIDMWSAGCVLAEL
251	LLGQPIFPGDSGVDQLVEIIKVLGTPTREQIREMNPNYTEFKFPQIKAHP
301	WTKVFRPRTPPEAIALCSRLLEYTPTARLTPLEACAHSFFDELRDPNVKL
351	PNGRDTPALFNFTTQELSSNPPLATILIPPHARIQAAAS**[P390]**PANATAASDT
401	N**[A402]**GDRGQTNNAASASASNST

Trypsin							
				Phospho Peptide		Non Phospho Peptide	
Peptide Sequence	Length	% hydro - phobic amino acids	Predicted Charge State(z)	Mono Isotpoic mass	Predicted m/z	Theoretical Mass	Predicted m/z
VTTVVA**[T43]**PGQGPDRPQEVSYTDTK	24	16.7	3.0	2625.2323	876.0847	2545.2660	849.4293
IQAAAS**[P390]**PANATAASDTN**[A402]**GDR	22	4.6	2.0	2228.9100	1115.4623	2068.9773	1035.4959
IQAAAS**[P390]**PANATAASDTNAGDR	22	4.6	2.0	2148.9437	1075.4791	2068.9773	1035.4959
IQAAASPPANATAASDTN**[A402]**GDR	22	4.6	2.0	2148.9437	1075.4791	2068.9773	1035.4959

Chymotrypsin-low specificity							
				Phospho Peptide		Non Phospho Peptide	
Peptide Sequence	Length	% hydro - phobic amino acids	Predicted Charge State(z)	Mono Isotpoic mass	Predicted m/z	Theoretical Mass	Predicted m/z
SGRPRTT**[S9]**F	9	11.1	3.0	1087.4812	363.5010	1007.5148	336.8456
KVSRDKDGSKVTTVVA**[T43]**PGQGPDRPQEVSY	30	16.7	6.0	3280.6088	547.7754	3200.6425	534.4477

Arg-C-proteinase							
				Phospho Peptide		Non Phospho Peptide	
Peptide Sequence	Length	% hydro - phobic amino acids	Predicted Charge State(z)	Mono Isotpoic mass	Predicted m/z	Theoretical Mass	Predicted m/z
DKDGSKVTTVVA**[T43]**PGQGPDR	20	15.0	4.0	2106.9946	527.7559	2027.0283	507.7644
IQAAAS**[P390]**PANATAASDTN**[A402]**GDR	22	4.6	2.0	2228.9100	1115.4623	2068.9773	1035.4959
IQAAAS**[P390]**PANATAASDTNAGDR	22	4.6	2.0	2148.9437	1075.4791	2068.9773	1035.4959
IQAAASPPANATAASDTN**[A402]**GDR	22	4.6	2.0	2148.9437	1075.4791	2068.9773	1035.4959

Asp-N endopeptidase							
				Phospho Peptide		Non Phospho Peptide	
Peptide Sequence	Length	% hydro - phobic amino acids	Predicted Charge State(z)	Mono Isotpoic mass	Predicted m/z	Theoretical Mass	Predicted m/z
DGSKVTTVVA**[T43]**PGQGP	16	18.8	2.0	1592.7447	797.3796	1512.7783	757.3965
DTN**[A402]**G	5	0.0	1.0	556.1530	557.1603	476.1867	477.1940

Clostripain (Clostridiopeptidase B)							
				Phospho Peptide		Non Phospho Peptide	
Peptide Sequence	Length	% hydro - phobic amino acids	Predicted Charge State(z)	Mono Isotpoic mass	Predicted m/z	Theoretical Mass	Predicted m/z
DKDGSKVTTVVA**[T43]**PGQGPDR	20	15.0	4.0	2106.9946	527.7559	2027.0283	507.7644
IQAAAS**[P390]**PANATAASDTN**[A402]**GDR	22	4.6	2.0	2228.9100	1115.4623	2068.9773	1035.4959
IQAAAS**[P390]**PANATAASDTNAGDR	22	4.6	2.0	2148.9437	1075.4791	2068.9773	1035.4959
IQAAASPPANATAASDTN**[A402]**GDR	22	4.6	2.0	2148.9437	1075.4791	2068.9773	1035.4959

Formic acid							
				Phospho Peptide		Non Phospho Peptide	
Peptide Sequence	Length	% hydro - phobic amino acids	Predicted Charge State(z)	Mono Isotpoic mass	Predicted m/z	Theoretical Mass	Predicted m/z
GSKVTTVVA**[T43]**PGQGPD	16	18.8	2.0	1592.7447	797.3796	1512.7783	757.3965
TN**[A402]**GD	5	0.0	1.0	556.1530	557.1603	476.1867	477.1940

LysC							
				Phospho Peptide		Non Phospho Peptide	
Peptide Sequence	Length	% hydro - phobic amino acids	Predicted Charge State(z)	Mono Isotpoic mass	Predicted m/z	Theoretical Mass	Predicted m/z
VTTVVA**[T43]**PGQGPDRPQEVSYTDTK	24	16.7	3.0	2625.2323	876.0847	2545.2660	849.4293

Proteinase K							
				Phospho Peptide		Non Phospho Peptide	
Peptide Sequence	Length	% hydro - phobic amino acids	Predicted Charge State(z)	Mono Isotpoic mass	Predicted m/z	Theoretical Mass	Predicted m/z
QQP**[S21]**A	5	0.0	1.0	609.2160	610.2232	529.2496	530.2569
A**[T43]**PGQGPDR PQE	12	0.0	2.0	1331.5507	666.7826	1251.5843	626.7995
N**[A402]**GDRGQT	8	0.0	2.0	897.3342	449.6744	817.3678	409.6912

Thermolysin							
				Phospho Peptide		Non Phospho Peptide	
Peptide Sequence	Length	% hydro - phobic amino acids	Predicted Charge State(z)	Mono Isotpoic mass	Predicted m/z	Theoretical Mass	Predicted m/z
VQQP**[S21]**AFGS	9	22.2	1.0	999.4063	1000.4135	919.4399	920.4472
A**[T43]**PGQGPDR PQEVSYTDTK	19	5.3	3.0	2125.9317	709.6512	2045.9654	682.9957
AASDTN**[A402]**G DRGQTNN	15	0.0	2.0	1570.6009	786.3077	1490.6346	746.3246

*P*Chopper

Experiment Name

Exp6

Search Term

Foxo1

Protein Name

Forkhead box protein O1A

Fragment Filter Criterion

No M

No C

Fragment Length Criterion

Between 5 and 30

Target Protein Sequence

```
1         MAEAPQVVEIDPDFEPLPRPRSC[T24]WPLPRPEFSQSNSATSSPAPSGSAAA
51        NPDAAAGLPSASAAAVSADFMSNLSLLEESEDFPQAPGSVAAAVAAAAAA
101       AATGGLCGDFQGPEAGCLHPAPPQPPPPGPLSQHPPVPPAAAGPLAGQPR
151       KSSSSRRNAWGNLSYADLITKAIESSAEKRLTLSQIYEWMVKSVPYFKDK
201       GDSNSSAGWKNSIRHNLSLHSKFIRVQNEGTGKSSWWMLNPEGGKSGKSP
251       RRRAA[S256]MDNNSKFAKSRSRAAKKKASLQSGQEGAGDSPGSQFSKWPASPG
301       SHSNDDFDNWSTFRPRTS[S319]NA[S322]TI[S325]GRL[S329]PIMTEQDDLGEGDVHSMVYPP
351       SAAKMASTLPSLSEISNPENMENLLDNLNLLSSPTSLTVSTQSSPGTMMQ
401       QTPCYSFAPPNTSLNSPSPNYQKYTYGQSSMSPLPQMPIQTLQDNKSSYG
451       GMSQYNCAPGLLKELLTSDSPPHNDIMTPVDPGVAQPNSRVLGQNVMMGP
501       NSVMSTYGSQASHNKMMNPSSHTHPGHAQQTSAVNGRPLPHTVSTMPHTS
551       GMNRLTQVKTPVQVPLPHPMQMSALGGYSSVSSCNGYGRMGLLHQEKLPS
601       DLDGMFIERLDCDMESIIRNDLMDGDTLDFNFDNVLPNQSFPHSVKTTTH
651       SWVSG
```

Trypsin							
				Phospho Peptide		Non Phospho Peptide	
Peptide Sequence	Length	% hydro - phobic amino acids	Predicted Charge State(z)	Mono Isotpoic mass	Predicted m/z	Theoretical Mass	Predicted m/z
TS**[S319]**NA**[S322]**TI**[S325]**GR	11	9.1	2.0	1319.4197	660.7171	1079.5207	540.7676
TS**[S319]**NASTISGR	11	9.1	2.0	1159.4870	580.7508	1079.5207	540.7676
TSSNA**[S322]**TISGR	11	9.1	2.0	1159.4870	580.7508	1079.5207	540.7676
TSSNASTI**[S325]**GR	11	9.1	2.0	1159.4870	580.7508	1079.5207	540.7676
TS**[S319]**NA**[S322]**TISGR	11	9.1	2.0	1239.4534	620.7340	1079.5207	540.7676
TS**[S319]**NASTI**[S325]**GR	11	9.1	2.0	1239.4534	620.7340	1079.5207	540.7676
TSSNA**[S322]**TI**[S325]**GR	11	9.1	2.0	1239.4534	620.7340	1079.5207	540.7676

Arg-C-proteinase							
				Phospho Peptide		Non Phospho Peptide	
Peptide Sequence	Length	% hydro - phobic amino acids	Predicted Charge State(z)	Mono Isotpoic mass	Predicted m/z	Theoretical Mass	Predicted m/z
TS**[S319]**NA**[S322]**TI**[S325]**GR	11	9.1	2.0	1319.4197	660.7171	1079.5207	540.7676
TS**[S319]**NASTISGR	11	9.1	2.0	1159.4870	580.7508	1079.5207	540.7676
TSSNA**[S322]**TISGR	11	9.1	2.0	1159.4870	580.7508	1079.5207	540.7676
TSSNASTI**[S325]**GR	11	9.1	2.0	1159.4870	580.7508	1079.5207	540.7676
TS**[S319]**NA**[S322]**TISGR	11	9.1	2.0	1239.4534	620.7340	1079.5207	540.7676
TS**[S319]**NASTI**[S325]**GR	11	9.1	2.0	1239.4534	620.7340	1079.5207	540.7676
TSSNA**[S322]**TI**[S325]**GR	11	9.1	2.0	1239.4534	620.7340	1079.5207	540.7676

Clostripain (Clostridiopeptidase B)							
				Phospho Peptide		Non Phospho Peptide	
Peptide Sequence	Length	% hydro - phobic amino acids	Predicted Charge State(z)	Mono Isotpoic mass	Predicted m/z	Theoretical Mass	Predicted m/z
TS[S319]NA[S322]TI[S325]GR	11	9.1	2.0	1319.4197	660.7171	1079.5207	540.7676
TS[S319]NASTISGR	11	9.1	2.0	1159.4870	580.7508	1079.5207	540.7676
TSSNA[S322]TISGR	11	9.1	2.0	1159.4870	580.7508	1079.5207	540.7676
TSSNASTI[S325]GR	11	9.1	2.0	1159.4870	580.7508	1079.5207	540.7676
TS[S319]NA[S322]TISGR	11	9.1	2.0	1239.4534	620.7340	1079.5207	540.7676
TS[S319]NASTI[S325]GR	11	9.1	2.0	1239.4534	620.7340	1079.5207	540.7676
TSSNA[S322]TI[S325]GR	11	9.1	2.0	1239.4534	620.7340	1079.5207	540.7676

Proteinase K							
				Phospho Peptide		Non Phospho Peptide	
Peptide Sequence	Length	% hydro - phobic amino acids	Predicted Charge State(z)	Mono Isotpoic mass	Predicted m/z	Theoretical Mass	Predicted m/z
S[S319]NA[S322]T	6	0.0	1.0	725.1670	726.1743	565.2344	566.2416
I[S325]GRL[S329]PI	8	37.5	2.0	1001.4348	501.7247	841.5021	421.7583
S[S319]NAST	6	0.0	1.0	645.2007	646.2080	565.2344	566.2416
SSNA[S322]T	6	0.0	1.0	645.2007	646.2080	565.2344	566.2416
I[S325]GRLSPI	8	37.5	2.0	921.4685	461.7415	841.5021	421.7583
ISGRL[S329]PI	8	37.5	2.0	921.4685	461.7415	841.5021	421.7583

Thermolysin							
				Phospho Peptide		Non Phospho Peptide	
Peptide Sequence	Length	% hydro - phobic amino acids	Predicted Charge State(z)	Mono Isotpoic mass	Predicted m/z	Theoretical Mass	Predicted m/z
FRPRTS[S319]N	8	12.5	3.0	1043.4550	348.8256	963.4886	322.1701

PChopper

Experiment Name

Exp7

Search Term

Tsc2

Protein Name

Tuberin

Fragment Filter Criterion

No M

No C

Fragment Length Criterion

Between 5 and 30

Target Protein Sequence

1 MAKPTSKDSGLKEKFKILLGLGTPRPNPRSAEGKQTEFIITAEILRELSM
51 ECGLNNRIRMIGQICEVAKTKKFEEHAVEALWKAVADLLQPERTLEARHA
101 VLALLKAIVQGQGERLGVLRALFFKVIKDYPSNEDLHERLEVFKALTDNG
151 RHITYLEEELADFVLQWMDVGLSSEFLLVLVNLVKFNSCYLDEYIARMVQ
201 MICLLCVRTASSVDIEVSLQVLDAVVCYNCLPAESLPLFIVTLCRTINVK
251 ELCEPCWKLMRNLLGTHLGHSAIYNMCHLMEDRAYMEDAPLLRGAVFFVG
301 MALWGAHRLYSLRNSPTSVFPSFYQAMACPNEVVSYEIVLSITRLIKKYR
351 KELQVVAWDILLNIIERLLQQLQTLDSPELRTIVHDLLTTVEELCDQNEF
401 HGSQERYFELVERCADQRPESSLLNLISYRAQSIHPAKDGWIQNLQALME
451 RFFRSESRGAVRIKVLDVLSFVLLINRQFYEEELINSVVISQLSHIPEDK
501 DHQVRKLATQLLVDLAEGCHTHHFNSLLDIIEKVMARSL**[S540]**PPPELEERDV
551 AAYSASLEDVKTAVLGLLVILQTKLYTLPASHATRVYEMLVSHIQLHYKH
601 SYTLPIASSIRLQAFDFLFLLRADSLHRLGLPNKDGVVRFSPYCVCDYME
651 PERGSEKKTSGPL**[S664]**PPTGPPGPAPAGPAVRLGSVPYSLLFRVLLQCLKQE
701 SDWKVLKLVLGRLPESLRYKVLIFTSPCSVDQLCSALCSMLSGPKTLERL
751 RGAPEGFSRTDLHLAVVPVLTALISYHNYLDKTKQREMVYCLEQGLIHRC
801 ARQCVVALSICSVEMPDIIIKALPVLVVKLTHISATASMAVPLLEFLSTL
851 ARLPHLYRNFAAEQYASVFAISLPYTNPSKFNQYIVCLAHHVIAMWFIRC
901 RLPFRKDFVPFITKGLRSNVLLSFDDTPEKDSFRARST**[S939]**LNERPKSLRIA
951 RPPKQGLNNSPPVKEFKESSAAEAFRCRSI**[S981]**VSEHVVRSRIQTSLTSASL
1001 GSADENSVAQADDSLKNLHLELTETCLDMMARYVFSNFTAVPKRSPVGEF
1051 LLAGGRTKTWLVGNKLVTVTTSVGTGTRSLLGLDSGELQSGPESSSSPGV
1101 HVRQTKEAPAKLESQAGQQVSRGARDRVRSMSGGHGLRVGALDVPASQFL
1151 GSATSPGPRTAPAAKPEKASAGTRVPVQEKTNLAAYVPLLTQGWAEILVR
1201 RPTGNTSWLMSLENPLSPFSSDINNMPLQELSNALMAAERFKEHRDTALY
1251 KSL**[S1254]**VPAASTAKPPPLPRSNTVASFSSLYQSSCQGQLHRSVSWADSAVVM
1301 EEGSPGEVPVLVEPPGLEDVEAALGMDRRTDAYSRSSSVSSQEEKSLHAE
1351 ELVGRGIPIERVVSSEGGRPSVDLSFQPSQPLSKSS**[S1387]**SPELQTLQDILGD
1401 PGDKADVGRL**[S1411]**PEVKAR**[S1418]**Q**[S1420]**GTLDGESAAWSASGEDSRGQPEGPLPSSSP
1451 RSPSGLRPRGY**[T1462]**ISDSAPSRRGKRVERDALKSRATASNAEKVPGINPSFV
1501 FLQLYHSPFFGDESNKPILLPNESQSFERSVQLLDQIPSYDTHKIAVLYV
1551 GEGQSNSELAILSNEHGSYR**[Y1571]**TEFLTGLGRLIELKDCQPDKVYLGGLDVC
1601 GEDGQFTYCWHDDIMQAVFHIATLMPTKDVDKHRCDKKRHLGNDFVSIVY
1651 NDSGEDFKLGTIKGQFNFVHVIVTPLDYECNLVSLQCRKDMEGLVDTSVA
1701 KIVSDRNLPFVARQMALHANMASQVHHSRSNPTDIYPSKWIARLRHIKRL
1751 RQRICEEAAYSNPSLPLVHPPSHSKAPAQTPAEPTPGYEVGQRKRLI**[S1798]**SV
1801 EDFTEFV

Trypsin							
				Phospho Peptide		Non Phospho Peptide	
Peptide Sequence	Length	% hydro - phobic amino acids	Predicted Charge State(z)	Mono Isotpoic mass	Predicted m/z	Theoretical Mass	Predicted m/z
SL**[S540]**PPPELEER	11	18.2	2.0	1332.5962	667.3054	1252.6299	627.3222
KTSGPL**[S664]**PPTGPPGPAPAGPAVR	23	8.7	3.0	2188.1041	730.3753	2108.1378	703.7199
ST**[S939]**LNERPK	9	11.1	3.0	1110.5070	371.1763	1030.5407	344.5208
SI**[S981]**VSEHVVR	10	40.0	3.0	1191.5649	398.1956	1111.5986	371.5401
SL**[S1254]**VPAASTAKPPPLPR	17	17.6	3.0	1767.9284	590.3167	1687.9621	563.6613
SS**[S1387]**SPELQTLQDILGDPGDK	20	20.0	2.0	2165.9729	1083.9937	2086.0066	1044.0106
L**[S1411]**PEVK	6	33.3	2.0	751.3517	376.6831	671.3854	336.7000
AR**[S1418]**Q**[S1420]**GTLDGESAAWSASGEDSR	23	8.7	3.0	2483.9591	828.9937	2324.0265	775.6828
GY**[T1462]**ISDSAPSR	11	9.1	2.0	1232.5074	617.2610	1152.5411	577.2778
LI**[S1798]**SVEDFTEFV	12	50.0	1.0	1464.6425	1465.6498	1384.6762	1385.6835
AR**[S1418]**QSGTLDGESAAWSASGEDSR	23	8.7	3.0	2403.9928	802.3382	2324.0265	775.6828
ARSQ**[S1420]**GTLDGESAAWSASGEDSR	23	8.7	3.0	2403.9928	802.3382	2324.0265	775.6828

Chymotrypsin-low specificity							
				Phospho Peptide		Non Phospho Peptide	
Peptide Sequence	Length	% hydro - phobic amino acids	Predicted Charge State(z)	Mono Isotpoic mass	Predicted m/z	Theoretical Mass	Predicted m/z
ARSL[S540]PPPEL	10	20.0	2.0	1145.5482	573.7814	1065.5818	533.7982
RARST[S939]L	7	14.3	3.0	869.4120	290.8113	789.4457	264.1558
YKSL[S1254]VPAASTAKPPPLPRSNTVASF	26	19.2	4.0	2765.4153	692.3611	2685.4490	672.3695
SKSS[S1387]SPEL	9	11.1	2.0	1000.4114	501.2130	920.4451	461.2298
GDPGDKADVGRL[S1411]PEVKAR[S1418]Q[S1420]GTL	25	16.0	5.0	2779.1980	556.8469	2539.2990	508.8671
RPRGY[T1462]ISDSAPSRRGKRVERDAL	24	12.5	8.0	2822.4413	353.8124	2742.4749	343.8166
R[Y1571]TEF	5	20.0	2.0	794.3000	398.1573	714.3337	358.1741
I[S1798]SVEDF	7	42.9	1.0	875.3314	876.3386	795.3650	796.3723
GDPGDKADVGRL[S1411]PEVKARSQSGTL	25	16.0	5.0	2619.2653	524.8603	2539.2990	508.8671
GDPGDKADVGRLSPEVKAR[S1418]QSGTL	25	16.0	5.0	2619.2653	524.8603	2539.2990	508.8671
GDPGDKADVGRLSPEVKARSQ[S1420]GTL	25	16.0	5.0	2619.2653	524.8603	2539.2990	508.8671
GDPGDKADVGRL[S1411]PEVKAR[S1418]QSGTL	25	16.0	5.0	2699.2316	540.8536	2539.2990	508.8671
GDPGDKADVGRL[S1411]PEVKARSQ[S1420]GTL	25	16.0	5.0	2699.2316	540.8536	2539.2990	508.8671
GDPGDKADVGRLSPEVKAR[S1418]Q[S1420]GTL	25	16.0	5.0	2699.2316	540.8536	2539.2990	508.8671

Chymotrypsin-high specificity							
				Phospho Peptide		Non Phospho Peptide	
Peptide Sequence	Length	% hydro - phobic amino acids	Predicted Charge State(z)	Mono Isotpoic mass	Predicted m/z	Theoretical Mass	Predicted m/z
KSL**[S1254]**VPAA STAKPPPLPRSNT VASF	25	20.0	4.0	2602.3520	651.5953	2522.3856	631.6037
R**[Y1571]**TEF	5	20.0	2.0	794.3000	398.1573	714.3337	358.1741
EVGQRKRLI**[S1798]**SVEDF	15	33.3	4.0	1841.9036	461.4832	1761.9373	441.4916

Arg-C-proteinase							
				Phospho Peptide		Non Phospho Peptide	
Peptide Sequence	Length	% hydro - phobic amino acids	Predicted Charge State(z)	Mono Isotpoic mass	Predicted m/z	Theoretical Mass	Predicted m/z
SL**[S540]**PPPELEER	11	18.2	2.0	1332.5962	667.3054	1252.6299	627.3222
GSEKKTSGPL**[S664]**PPTGPPGPAPAGPAVR	27	7.4	4.0	2589.2951	648.3311	2509.3288	628.3395
ST**[S939]**LNER	7	14.3	2.0	885.3593	443.6869	805.3930	403.7038
SI**[S981]**VSEHVVR	10	40.0	3.0	1191.5649	398.1956	1111.5986	371.5401
DTALYKSL**[S1254]**VPAASTAKPPPLPR	23	17.4	4.0	2459.2825	615.8279	2379.3161	595.8363
L**[S1411]**PEVKAR**[S1418]**Q**[S1420]**GTLDGESAAWSASGEDSR	29	13.8	4.0	3217.3003	805.3323	2977.4013	745.3576
GY**[T1462]**ISDSAPSR	11	9.1	2.0	1232.5074	617.2610	1152.5411	577.2778
LI**[S1798]**SVEDFTEFV	12	50.0	1.0	1464.6425	1465.6498	1384.6762	1385.6835
L**[S1411]**PEVKARSQSGTLDGESAAWSASGEDSR	29	13.8	4.0	3057.3676	765.3492	2977.4013	745.3576
LSPEVKAR**[S1418]**QSGTLDGESAAWSASGEDSR	29	13.8	4.0	3057.3676	765.3492	2977.4013	745.3576
LSPEVKARSQ**[S1420]**GTLDGESAAWSASGEDSR	29	13.8	4.0	3057.3676	765.3492	2977.4013	745.3576
L**[S1411]**PEVKAR**[S1418]**QSGTLDGESAAWSASGEDSR	29	13.8	4.0	3137.3339	785.3408	2977.4013	745.3576
L**[S1411]**PEVKARSQ**[S1420]**GTLDGESAAWSASGEDSR	29	13.8	4.0	3137.3339	785.3408	2977.4013	745.3576
LSPEVKAR**[S1418]**Q**[S1420]**GTLDGESAAWSASGEDSR	29	13.8	4.0	3137.3339	785.3408	2977.4013	745.3576

Asp-N endopeptidase							
				Phospho Peptide		Non Phospho Peptide	
Peptide Sequence	Length	% hydro - phobic amino acids	Predicted Charge State(z)	Mono Isotpoic mass	Predicted m/z	Theoretical Mass	Predicted m/z
DLSFQPSQPLSKSS**[S1387]**SPELQTLQ	23	21.7	2.0	2583.2105	1292.6125	2503.2442	1252.6294
DVGRL**[S1411]**PEVKAR**[S1418]**Q**[S1420]**GTL	18	22.2	4.0	2138.9163	535.7364	1899.0173	475.7616
DSRGQPEGPLPSSSPRSPSGLRPRGY**[T1462]**IS	29	10.3	5.0	3117.4992	624.5071	3037.5329	608.5139
DVGRL**[S1411]**PEVKARSQSGTL	18	22.2	4.0	1978.9837	495.7532	1899.0173	475.7616
DVGRLSPEVKAR**[S1418]**QSGTL	18	22.2	4.0	1978.9837	495.7532	1899.0173	475.7616
DVGRLSPEVKARSQ**[S1420]**GTL	18	22.2	4.0	1978.9837	495.7532	1899.0173	475.7616
DVGRL**[S1411]**PEVKAR**[S1418]**QSGTL	18	22.2	4.0	2058.9500	515.7448	1899.0173	475.7616
DVGRL**[S1411]**PEVKARSQ**[S1420]**GTL	18	22.2	4.0	2058.9500	515.7448	1899.0173	475.7616
DVGRLSPEVKAR**[S1418]**Q**[S1420]**GTL	18	22.2	4.0	2058.9500	515.7448	1899.0173	475.7616

Clostripain (Clostridiopeptidase B)							
				Phospho Peptide		Non Phospho Peptide	
Peptide Sequence	Length	% hydro - phobic amino acids	Predicted Charge State(z)	Mono Isotpoic mass	Predicted m/z	Theoretical Mass	Predicted m/z
SL**[S540]**PPPELEER	11	18.2	2.0	1332.5962	667.3054	1252.6299	627.3222
GSEKKTSGPL**[S664]**PPTGPPGPAPAGPAVR	27	7.4	4.0	2589.2951	648.3311	2509.3288	628.3395
ST**[S939]**LNER	7	14.3	2.0	885.3593	443.6869	805.3930	403.7038
SI**[S981]**VSEHVVR	10	40.0	3.0	1191.5649	398.1956	1111.5986	371.5401
DTALYKSL**[S1254]**VPAASTAKPPPLPR	23	17.4	4.0	2459.2825	615.8279	2379.3161	595.8363
L**[S1411]**PEVKAR**[S1418]**Q**[S1420]**GTLDGESAAWSASGEDSR	29	13.8	4.0	3217.3003	805.3323	2977.4013	745.3576
GY**[T1462]**ISDSAPSR	11	9.1	2.0	1232.5074	617.2610	1152.5411	577.2778
LI**[S1798]**SVEDFTEFV	12	50.0	1.0	1464.6425	1465.6498	1384.6762	1385.6835
L**[S1411]**PEVKARSQSGTLDGESAAWSASGEDSR	29	13.8	4.0	3057.3676	765.3492	2977.4013	745.3576
LSPEVKAR**[S1418]**QSGTLDGESAAWSASGEDSR	29	13.8	4.0	3057.3676	765.3492	2977.4013	745.3576
LSPEVKARSQ**[S1420]**GTLDGESAAWSASGEDSR	29	13.8	4.0	3057.3676	765.3492	2977.4013	745.3576
L**[S1411]**PEVKAR**[S1418]**QSGTLDGESAAWSASGEDSR	29	13.8	4.0	3137.3339	785.3408	2977.4013	745.3576
L**[S1411]**PEVKARSQ**[S1420]**GTLDGESAAWSASGEDSR	29	13.8	4.0	3137.3339	785.3408	2977.4013	745.3576
LSPEVKAR**[S1418]**Q**[S1420]**GTLDGESAAWSASGEDSR	29	13.8	4.0	3137.3339	785.3408	2977.4013	745.3576

Formic acid							
				Phospho Peptide		Non Phospho Peptide	
Peptide Sequence	Length	% hydro - phobic amino acids	Predicted Charge State(z)	Mono Isotpoic mass	Predicted m/z	Theoretical Mass	Predicted m/z
LSFQPSQPLSKSS**[S1387]**SPELQTLQD	23	21.7	2.0	2583.2105	1292.6125	2503.2442	1252.6294
VGRL**[S1411]**PEVKAR**[S1418]**Q**[S1420]**GTLD	18	22.2	4.0	2138.9163	535.7364	1899.0173	475.7616
SRGQPEGPLPSSSPRSPSGLRPRGY**[T1462]**ISD	29	10.3	5.0	3117.4992	624.5071	3037.5329	608.5139
VGRL**[S1411]**PEVKARSQSGTLD	18	22.2	4.0	1978.9837	495.7532	1899.0173	475.7616
VGRLSPEVKAR**[S1418]**QSGTLD	18	22.2	4.0	1978.9837	495.7532	1899.0173	475.7616
VGRLSPEVKARSQ**[S1420]**GTLD	18	22.2	4.0	1978.9837	495.7532	1899.0173	475.7616
VGRL**[S1411]**PEVKAR**[S1418]**QSGTLD	18	22.2	4.0	2058.9500	515.7448	1899.0173	475.7616
VGRL**[S1411]**PEVKARSQ**[S1420]**GTLD	18	22.2	4.0	2058.9500	515.7448	1899.0173	475.7616
VGRLSPEVKAR**[S1418]**Q**[S1420]**GTLD	18	22.2	4.0	2058.9500	515.7448	1899.0173	475.7616

Glutamyl endopeptidase							
				Phospho Peptide		Non Phospho Peptide	
Peptide Sequence	Length	% hydro - phobic amino acids	Predicted Charge State(z)	Mono Isotpoic mass	Predicted m/z	Theoretical Mass	Predicted m/z
KDSFRARST**[S939]**LNE	13	15.4	4.0	1589.7199	398.4372	1509.7536	378.4457
GGRPSVDLSFQPSQPLSKSS**[S1387]**SPE	24	16.7	3.0	2553.1748	852.0655	2473.2085	825.4101
LQTLQDILGDPGDKADVGRL**[S1411]**PE	23	26.1	3.0	2516.2159	839.7459	2436.2496	813.0905
VKAR**[S1418]**Q**[S1420]**GTLDGE	13	15.4	3.0	1506.6116	503.2112	1346.6790	449.9003
HGSYR**[Y1571]**TE	8	0.0	3.0	1091.4073	364.8097	1011.4410	338.1543
VGQRKRLI**[S1798]**SVE	12	33.3	4.0	1450.7657	363.6987	1370.7994	343.7071
VKAR**[S1418]**QSGTLDGE	13	15.4	3.0	1426.6453	476.5557	1346.6790	449.9003
VKARSQ**[S1420]**GTLDGE	13	15.4	3.0	1426.6453	476.5557	1346.6790	449.9003

LysC							
				Phospho Peptide		Non Phospho Peptide	
Peptide Sequence	Length	% hydro - phobic amino acids	Predicted Charge State(z)	Mono Isotpoic mass	Predicted m/z	Theoretical Mass	Predicted m/z
DSFRARST**[S939]**LNERPK	15	13.3	5.0	1842.8738	369.5820	1762.9074	353.5888
SL**[S1254]**VPAASTAK	11	18.2	2.0	1110.5322	556.2734	1030.5658	516.2902
SS**[S1387]**SPELQTLQDILGDPGDK	20	20.0	2.0	2165.9729	1083.9937	2086.0066	1044.0106
ADVGRL**[S1411]**PEVK	11	27.3	3.0	1249.6067	417.5429	1169.6404	390.8874
RLI**[S1798]**SVEDFTEFV	13	46.2	2.0	1620.7436	811.3791	1540.7773	771.3959

Pepsin (pH1.3)							
				Phospho Peptide		Non Phospho Peptide	
Peptide Sequence	Length	% hydro - phobic amino acids	Predicted Charge State(z)	Mono Isotpoic mass	Predicted m/z	Theoretical Mass	Predicted m/z
L**[S540]**PPPEL	7	28.6	1.0	831.3779	832.3852	751.4116	752.4189
L**[S664]**PPTGPPGPAPAGPAVRL	19	15.8	2.0	1830.9393	916.4769	1750.9729	876.4937
RARST**[S939]**L	7	14.3	3.0	869.4120	290.8113	789.4457	264.1558
YKSL**[S1254]**VPAASTAKPPPLPRSNTVAS	25	16.0	4.0	2618.3469	655.5940	2538.3805	635.6024
LSKSS**[S1387]**SPEL	10	20.0	2.0	1113.4955	557.7550	1033.5291	517.7718
GDPGDKADVGRL**[S1411]**PEVKAR**[S1418]**Q**[S1420]**GT	24	12.5	5.0	2666.1139	534.2301	2426.2149	486.2503
Y**[T1462]**ISDSAPSRRGKRVERDAL	20	15.0	6.0	2356.1648	393.7014	2276.1985	380.3737
EVGQRKRLI**[S1798]**SVED	14	28.6	4.0	1694.8352	424.7161	1614.8689	404.7245
GDPGDKADVGRL**[S1411]**PEVKARSQSGT	24	12.5	5.0	2506.1813	502.2435	2426.2149	486.2503
GDPGDKADVGRLSPEVKAR**[S1418]**QSGT	24	12.5	5.0	2506.1813	502.2435	2426.2149	486.2503
GDPGDKADVGRLSPEVKARSQ**[S1420]**GT	24	12.5	5.0	2506.1813	502.2435	2426.2149	486.2503
GDPGDKADVGRL**[S1411]**PEVKAR**[S1418]**QSGT	24	12.5	5.0	2586.1476	518.2368	2426.2149	486.2503
GDPGDKADVGRL**[S1411]**PEVKARSQ**[S1420]**GT	24	12.5	5.0	2586.1476	518.2368	2426.2149	486.2503
GDPGDKADVGRLSPEVKAR**[S1418]**Q**[S1420]**GT	24	12.5	5.0	2586.1476	518.2368	2426.2149	486.2503

Pepsin (pH1.2)							
				Phospho Peptide		Non Phospho Peptide	
Peptide Sequence	Length	% hydro - phobic amino acids	Predicted Charge State(z)	Mono Isotpoic mass	Predicted m/z	Theoretical Mass	Predicted m/z
L**[S540]**PPPEL	7	28.6	1.0	831.3779	832.3852	751.4116	752.4189
L**[S664]**PPTGPPGPAPAGPAVRL	19	15.8	2.0	1830.9393	916.4769	1750.9729	876.4937
RARST**[S939]**L	7	14.3	3.0	869.4120	290.8113	789.4457	264.1558
YKSL**[S1254]**VPAASTAKPPPLPRSNTVAS	25	16.0	4.0	2618.3469	655.5940	2538.3805	635.6024
LSKSS**[S1387]**SPEL	10	20.0	2.0	1113.4955	557.7550	1033.5291	517.7718
GDPGDKADVGRL**[S1411]**PEVKAR**[S1418]**Q**[S1420]**GT	24	12.5	5.0	2666.1139	534.2301	2426.2149	486.2503
LRPRGY**[T1462]**ISDSAPSRRGKRVERDAL	25	16.0	8.0	2935.5253	367.9479	2855.5590	357.9522
AILSNEHGSYR**[Y1571]**TE	14	14.3	3.0	1718.7301	573.9173	1638.7638	547.2619
GDPGDKADVGRL**[S1411]**PEVKARSQSGT	24	12.5	5.0	2506.1813	502.2435	2426.2149	486.2503
GDPGDKADVGRLSPEVKAR**[S1418]**QSGT	24	12.5	5.0	2506.1813	502.2435	2426.2149	486.2503
GDPGDKADVGRLSPEVKARSQ**[S1420]**GT	24	12.5	5.0	2506.1813	502.2435	2426.2149	486.2503
GDPGDKADVGRL**[S1411]**PEVKAR**[S1418]**QSGT	24	12.5	5.0	2586.1476	518.2368	2426.2149	486.2503
GDPGDKADVGRL**[S1411]**PEVKARSQ**[S1420]**GT	24	12.5	5.0	2586.1476	518.2368	2426.2149	486.2503
GDPGDKADVGRLSPEVKAR**[S1418]**Q**[S1420]**GT	24	12.5	5.0	2586.1476	518.2368	2426.2149	486.2503

Proteinase K							
				Phospho Peptide		Non Phospho Peptide	
Peptide Sequence	Length	% hydro - phobic amino acids	Predicted Charge State(z)	Mono Isotpoic mass	Predicted m/z	Theoretical Mass	Predicted m/z
RSL**[S540]**PPPE	8	12.5	2.0	961.4270	481.7208	881.4607	441.7376
SGPL**[S664]**PPT	8	12.5	1.0	834.3524	835.3597	754.3861	755.3934
RST**[S939]**L	5	20.0	2.0	642.2738	322.1442	562.3075	282.1610
KSL**[S1254]**V	5	40.0	2.0	612.2884	307.1515	532.3221	267.1683
SKSS**[S1387]**SPE	8	0.0	2.0	887.3274	444.6710	807.3610	404.6878
GRL**[S1411]**PE	6	16.7	2.0	737.3109	369.6627	657.3446	329.6796
R**[S1418]**Q**[S1420]**GT	6	0.0	2.0	794.2361	398.1253	634.3035	318.1590
RPRGY**[T1462]**I	7	14.3	3.0	941.4484	314.8234	861.4821	288.1680
R**[S1418]**QSGT	6	0.0	2.0	714.2698	358.1422	634.3035	318.1590
RSQ**[S1420]**GT	6	0.0	2.0	714.2698	358.1422	634.3035	318.1590

Staphylococcal peptidase I							
				Phospho Peptide		Non Phospho Peptide	
Peptide Sequence	Length	% hydro - phobic amino acids	Predicted Charge State(z)	Mono Isotpoic mass	Predicted m/z	Theoretical Mass	Predicted m/z
KDSFRARST**[S939]**LNE	13	15.4	4.0	1589.7199	398.4372	1509.7536	378.4457
GGRPSVDLSFQPSQPLSKSS**[S1387]**SPE	24	16.7	3.0	2553.1748	852.0655	2473.2085	825.4101
LQTLQDILGDPGDKADVGRL**[S1411]**PE	23	26.1	3.0	2516.2159	839.7459	2436.2496	813.0905
VKAR**[S1418]**Q**[S1420]**GTLDGE	13	15.4	3.0	1506.6116	503.2112	1346.6790	449.9003
HGSYR**[Y1571]**TE	8	0.0	3.0	1091.4073	364.8097	1011.4410	338.1543
VGQRKRLI**[S1798]**SVE	12	33.3	4.0	1450.7657	363.6987	1370.7994	343.7071
VKAR**[S1418]**QSGTLDGE	13	15.4	3.0	1426.6453	476.5557	1346.6790	449.9003
VKARSQ**[S1420]**GTLDGE	13	15.4	3.0	1426.6453	476.5557	1346.6790	449.9003

Thermolysin							
				Phospho Peptide		Non Phospho Peptide	
Peptide Sequence	Length	% hydro - phobic amino acids	Predicted Charge State(z)	Mono Isotpoic mass	Predicted m/z	Theoretical Mass	Predicted m/z
L**[S540]**PPPELEERDV	12	25.0	2.0	1459.6596	730.8371	1379.6932	690.8539
L**[S664]**PPTGPPGP	10	10.0	1.0	998.4474	999.4547	918.4811	919.4883
ARST**[S939]**LNERPKS	12	8.3	4.0	1424.6773	357.1766	1344.7110	337.1850
I**[S981]**VSEH	6	33.3	2.0	750.2949	376.1547	670.3286	336.1716
LSKSS**[S1387]**SPELQT	12	16.7	2.0	1342.6017	672.3081	1262.6354	632.3250
L**[S1411]**PEVK	6	33.3	2.0	751.3517	376.6831	671.3854	336.7000
AR**[S1418]**Q**[S1420]**GT	7	0.0	2.0	865.2732	433.6439	705.3406	353.6776
LRPRGY**[T1462]**ISDS	11	18.2	3.0	1343.6235	448.8818	1263.6571	422.2263
LSNEHGSYR**[Y1571]**TEF	13	15.4	3.0	1681.6774	561.5664	1601.7110	534.9109
AR**[S1418]**QSGT	7	0.0	2.0	785.3069	393.6607	705.3406	353.6776
ARSQ**[S1420]**GT	7	0.0	2.0	785.3069	393.6607	705.3406	353.6776

PChopper

Experiment Name

Exp8

Search Term

Mapk3

Protein Name

Mitogen-activated protein kinase 3

Fragment Filter Criterion

No M

No C

Fragment Length Criterion

Between 5 and 30

Target Protein Sequence

```
1       MAAAAAQGGGGGEPRRTEGVGPGVPGEVEMVKGQPFDVGPRYTQLQYIGE
51      GAYGMVSSAYDHVRKTRVAIKKISPFEHQTYCQRTLREIQILLRFRHENV
101     IGIRDILRASTLEAMRDVYIVQDLMETDLYKLLKSQQLSNDHICYFLYQI
151     LRGLKYIHSANVLHRDLKPSNLLINTTCDLKICDFGLARIADPEHDHTGF
201     L[T202]E[Y204]VATRWYRAPEIMLNSKGYTKSIDIWSVGCILAEMLSNRPIFPGKHY
251     LDQLNHILGILGSPSQEDLNCIINMKARNYLQSLPSKTKVAWAKLFPKSD
301     SKALDLLDRMLTFNPNKRITVEEALAHPYLEQYYDPTDEPVAEEPFTFAM
351     ELDDLPKERLKELIFQETARFQPGVLEAP
```

Trypsin							
				Phospho Peptide		Non Phospho Peptide	
Peptide Sequence	Length	% hydro - phobic amino acids	Predicted Charge State(z)	Mono Isotpoic mass	Predicted m/z	Theoretical Mass	Predicted m/z
IADPEHDHTGFL**[T202]**E**[Y204]**VATR	19	21.0	4.0	2330.9610	583.7475	2171.0283	543.7644
IADPEHDHTGFL**[T202]**EYVATR	19	21.0	4.0	2250.9946	563.7559	2171.0283	543.7644
IADPEHDHTGFLTE**[Y204]**VATR	19	21.0	4.0	2250.9946	563.7559	2171.0283	543.7644

Chymotrypsin-low specificity							
				Phospho Peptide		Non Phospho Peptide	
Peptide Sequence	Length	% hydro - phobic amino acids	Predicted Charge State(z)	Mono Isotpoic mass	Predicted m/z	Theoretical Mass	Predicted m/z
L**[T202]**E**[Y204]**VATRW	9	33.3	2.0	1297.5145	649.7645	1137.5818	569.7982
L**[T202]**EYVATRW	9	33.3	2.0	1217.5482	609.7814	1137.5818	569.7982
LTE**[Y204]**VATRW	9	33.3	2.0	1217.5482	609.7814	1137.5818	569.7982

Chymotrypsin-high specificity							
				Phospho Peptide		Non Phospho Peptide	
Peptide Sequence	Length	% hydro - phobic amino acids	Predicted Charge State(z)	Mono Isotpoic mass	Predicted m/z	Theoretical Mass	Predicted m/z
L**[T202]**E**[Y204]**VATRW	9	33.3	2.0	1297.5145	649.7645	1137.5818	569.7982
L**[T202]**EYVATRW	9	33.3	2.0	1217.5482	609.7814	1137.5818	569.7982
LTE**[Y204]**VATRW	9	33.3	2.0	1217.5482	609.7814	1137.5818	569.7982

Arg-C-proteinase							
				Phospho Peptide		Non Phospho Peptide	
Peptide Sequence	Length	% hydro - phobic amino acids	Predicted Charge State(z)	Mono Isotpoic mass	Predicted m/z	Theoretical Mass	Predicted m/z
IADPEHDHTGFL**[T202]**E**[Y204]**VATR	19	21.0	4.0	2330.9610	583.7475	2171.0283	543.7644
IADPEHDHTGFL**[T202]**EYVATR	19	21.0	4.0	2250.9946	563.7559	2171.0283	543.7644
IADPEHDHTGFLTE**[Y204]**VATR	19	21.0	4.0	2250.9946	563.7559	2171.0283	543.7644

Clostripain (Clostridiopeptidase B)							
				Phospho Peptide		Non Phospho Peptide	
Peptide Sequence	Length	% hydro - phobic amino acids	Predicted Charge State(z)	Mono Isotpoic mass	Predicted m/z	Theoretical Mass	Predicted m/z
IADPEHDHTGFL**[T202]**E**[Y204]**VATR	19	21.0	4.0	2330.9610	583.7475	2171.0283	543.7644
IADPEHDHTGFL**[T202]**EYVATR	19	21.0	4.0	2250.9946	563.7559	2171.0283	543.7644
IADPEHDHTGFLTE**[Y204]**VATR	19	21.0	4.0	2250.9946	563.7559	2171.0283	543.7644

Glutamyl endopeptidase							
				Phospho Peptide		Non Phospho Peptide	
Peptide Sequence	Length	% hydro - phobic amino acids	Predicted Charge State(z)	Mono Isotpoic mass	Predicted m/z	Theoretical Mass	Predicted m/z
HDHTGFL**[T202]**E**[Y204]**VATRWYRAPE	20	20.0	5.0	2608.0937	522.6260	2448.1611	490.6395
HDHTGFL**[T202]**EYVATRWYRAPE	20	20.0	5.0	2528.1274	506.6328	2448.1611	490.6395
HDHTGFLTE**[Y204]**VATRWYRAPE	20	20.0	5.0	2528.1274	506.6328	2448.1611	490.6395

Pepsin (pH1.3)							
				Phospho Peptide		Non Phospho Peptide	
Peptide Sequence	Length	% hydro - phobic amino acids	Predicted Charge State(z)	Mono Isotpoic mass	Predicted m/z	Theoretical Mass	Predicted m/z
L**[T202]**E**[Y204]**VATRW	9	33.3	2.0	1297.5145	649.7645	1137.5818	569.7982
L**[T202]**EYVATRW	9	33.3	2.0	1217.5482	609.7814	1137.5818	569.7982
LTE**[Y204]**VATRW	9	33.3	2.0	1217.5482	609.7814	1137.5818	569.7982

Proteinase K							
				Phospho Peptide		Non Phospho Peptide	
Peptide Sequence	Length	% hydro - phobic amino acids	Predicted Charge State(z)	Mono Isotpoic mass	Predicted m/z	Theoretical Mass	Predicted m/z
L**[T202]**E**[Y204]**V	5	40.0	1.0	783.2493	784.2566	623.3166	624.3239
L**[T202]**EYV	5	40.0	1.0	703.2830	704.2902	623.3166	624.3239
LTE**[Y204]**V	5	40.0	1.0	703.2830	704.2902	623.3166	624.3239

Staphylococcal peptidase I							
				Phospho Peptide		Non Phospho Peptide	
Peptide Sequence	Length	% hydro - phobic amino acids	Predicted Charge State(z)	Mono Isotpoic mass	Predicted m/z	Theoretical Mass	Predicted m/z
HDHTGFL**[T202]**E**[Y204]**VATRWYRAPE	20	20.0	5.0	2608.0937	522.6260	2448.1611	490.6395
HDHTGFL**[T202]**EYVATRWYRAPE	20	20.0	5.0	2528.1274	506.6328	2448.1611	490.6395
HDHTGFLTE**[Y204]**VATRWYRAPE	20	20.0	5.0	2528.1274	506.6328	2448.1611	490.6395

Thermolysin							
				Phospho Peptide		Non Phospho Peptide	
Peptide Sequence	Length	% hydro - phobic amino acids	Predicted Charge State(z)	Mono Isotpoic mass	Predicted m/z	Theoretical Mass	Predicted m/z
FL**[T202]**E**[Y204]**VATRWYR	12	33.3	3.0	1763.7474	588.9231	1603.8147	535.6122
FL**[T202]**EYVATRWYR	12	33.3	3.0	1683.7810	562.2676	1603.8147	535.6122
FLTE**[Y204]**VATRWYR	12	33.3	3.0	1683.7810	562.2676	1603.8147	535.6122

PChopper

Experiment Name

Exp9

Search Term

IRS1

Protein Name

Insulin receptor substrate 1

Fragment Filter Criterion

No M

No C

Fragment Length Criterion

Between 5 and 30

Target Protein Sequence

```
1       MASPPESDGFSDVRKVGYLRKPK[S24]MHKRFFVLRAASEAGGPARLE[Y46]YENE
51      KKWRHKSSAPKRSIPLESCFNINKRADSKNKHLVALYTRDEHFAIAADSE
101     AEQDSWYQALLQLHNRAKGHHDGAAALGAGGGGGSCSGSSGLGEAGEDLS
151     YGDVPPGPAFKEVWQVILKPKGLGQTKNLIGIYRLCLTSKTISFVKLNSE
201     AAAVVLQLMNIRRCGHSENFFFIEVGRSAVTGPGEFWMQVDDSVVAQNMH
251     ETILEAMRAMSDEFRPR[S268]K[S270]Q[S272]S[S274]NCSNPISVPLRRHHLNNPPPSQVGLT
301
RRSRTE[S307]ITAT[S312]PASMVGGKPG[S323]FRVRASSDGEGTMSRPA[S341]VDG[S345]PV[S348]PS
351     TNRTHAHRHRGSARLHPPLNHSR[S374]IPMPASRCSPSATSPVSLSSSSTSGH
401     GSTSDCLFPRRSSASVSGSPSDGGFISSDEYGSSPCDFRSSFRSVTPDSL
451     GHTPPARGEEELSNYICMGGKGPSTLTAPNGHYILSRGGNGHRCTPGTGL
501     GTSPALAGDEAASAADLDNRFRKRTH[S527]AG[T530][S531]PTITHQKTPSQSSVASIEE
551     YTEMMPAYPPGGGSGGRLPGHRH[S574]AFVPTRSYPEEGLEMHPLERRGGHHR
601
PDSSTLHTDDG[Y612]MPM[S616]PGVAPVPSGRKG[S629]GD[Y632]MPM[S636]PK[S639]VSAPQQIINPI
651     RRHPQRVDPNG[Y662]MMMSPSGGCSPDIGGGPSSSSSSSNAVPSGTSYGKLWT
701     NGVGGHHSHVLPHPKPPVESSGGKLLPCTGDYMNMSPVGDSNTSSPSDCY
751     YGPEDPQHKPVLSYYSLPRSFKHTQRPGEPEEGARHQHLRLST[S794]SGRLLY
801     AATADDSSSSTSSDSLGGGYCGARLEPSLPHPHHQVLQPHLPRKVDTAAQ
851     TNSRLARPTRLSLGDPKASTLPRAREQQQQQQPLLHPPEPK[S892]PGE[Y896]VNIE
901     FGSDQSGYLSGPVAFHSSPSVRCPSQLQPAPREEETGTEEYMKMDLGPGR
951     RAAWQESTGVEMGRLGPAPPGAASICRPTRAVPSSRGDYMTMQMSCPRQS
1001    YVDTSPAAPVSYADMRTGIAAEEVSLPRATMAAASSSSAASASPTGPQGA
1051    AELAAHSSLLGGPQGPGGMSAFTRVNL[S1078]PNRNQSAKVIRADPQGCRRRHS
1101    [S1101]ETFSSTPSATRVGNTVPFGAGAAVGGGGGSSSSSEDVKRHSSA[S1145]FENVW
1151    LRPGELGGAPKEPAKLCGAAGGLENGLNYIDLDVKDFKQCPQECTPEPQ
1201    PPPPPPPHQPLGSGESSSTRR[S1222][S1223]EDLSAYASISFQKQPEDRQ
```

Trypsin							
				Phospho Peptide		Non Phospho Peptide	
Peptide Sequence	Length	% hydro - phobic amino acids	Predicted Charge State(z)	Mono Isotpoic mass	Predicted m/z	Theoretical Mass	Predicted m/z
LE**[Y46]**YENEK	8	12.5	2.0	1166.4533	584.2339	1086.4869	544.2507
TH**[S527]**AG**[T530][S531]**PTITHQK	14	7.1	4.0	1704.6311	427.1650	1464.7321	367.1903
H**[S574]**AFVPTR	8	25.0	3.0	993.4433	332.1550	913.4770	305.4996
LST**[S794]**SGR	7	14.3	2.0	786.3273	394.1709	706.3610	354.1878
VNL**[S1078]**PNR	7	28.6	2.0	878.4011	440.2078	798.4348	400.2247
HS**[S1101]**ETFSSTPSATR	14	7.1	3.0	1573.6410	525.5543	1493.6746	498.8988
RHSSA**[S1145]**FENVWLRPGELGGAPK	22	22.7	5.0	2474.1856	495.8444	2394.2192	479.8511
R**[S1222][S1223]**EDLSAYASISFQK	16	18.8	3.0	1947.8016	650.2745	1787.8690	596.9636
TH**[S527]**AGTSPTITHQK	14	7.1	4.0	1544.6984	387.1819	1464.7321	367.1903
THSAG**[T530]**SPTITHQK	14	7.1	4.0	1544.6984	387.1819	1464.7321	367.1903
THSAGT**[S531]**PTITHQK	14	7.1	4.0	1544.6984	387.1819	1464.7321	367.1903
TH**[S527]**AG**[T530]**SPTITHQK	14	7.1	4.0	1624.6647	407.1735	1464.7321	367.1903
TH**[S527]**AGT**[S531]**PTITHQK	14	7.1	4.0	1624.6647	407.1735	1464.7321	367.1903
THSAG**[T530][S531]**PTITHQK	14	7.1	4.0	1624.6647	407.1735	1464.7321	367.1903
R**[S1222]**SEDLSAYASISFQK	16	18.8	3.0	1867.8353	623.6190	1787.8690	596.9636
RS**[S1223]**EDLSAYASISFQK	16	18.8	3.0	1867.8353	623.6190	1787.8690	596.9636

Chymotrypsin-low specificity							
				Phospho Peptide		Non Phospho Peptide	
Peptide Sequence	Length	% hydro - phobic amino acids	Predicted Charge State(z)	Mono Isotpoic mass	Predicted m/z	Theoretical Mass	Predicted m/z
E**[Y46]**YENEKKW	9	11.1	3.0	1367.5435	456.8551	1287.5771	430.1997
VGGKPG**[S323]**F	8	25.0	2.0	827.3578	414.6862	747.3915	374.7030
SRPA**[S341]**VDG**[S345]**PV**[S348]**PSTNRTHAHRHRGSARL	29	10.3	9.0	3332.4827	371.2831	3092.5837	344.6277
RKRTH**[S527]**AG**[T530][S531]**PTITHQKTPSQSSVASIEEY	30	10.0	7.0	3523.5535	504.3721	3283.6545	470.1008
ST**[S794]**SGRL	7	14.3	2.0	786.3273	394.1709	706.3610	354.1878
PPEPK**[S892]**PGE**[Y896]**VNIEF	15	20.0	2.0	1861.7576	931.8861	1701.8250	851.9198
GAGAAVGGGGGSSSSSEDVKRHSSA**[S1145]**F	27	11.1	4.0	2488.0615	623.0227	2408.0952	603.0311
GSGESSSTRR**[S1222][S1223]**EDL	15	6.7	3.0	1713.6244	572.2154	1553.6917	518.9045
SRPA**[S341]**VDGSPVSPSTNRTHAHRHRGSARL	29	10.3	9.0	3172.5500	353.5128	3092.5837	344.6277
SRPASVDG**[S345]**PVSPSTNRTHAHRHRGSARL	29	10.3	9.0	3172.5500	353.5128	3092.5837	344.6277
SRPASVDGSPV**[S348]**PSTNRTHAHRHRGSARL	29	10.3	9.0	3172.5500	353.5128	3092.5837	344.6277
SRPA**[S341]**VDG**[S345]**PVSPSTNRTHAHRHRGSARL	29	10.3	9.0	3252.5163	362.3980	3092.5837	344.6277
SRPA**[S341]**VDGSPV**[S348]**PSTNRTHAHRHRGSARL	29	10.3	9.0	3252.5163	362.3980	3092.5837	344.6277
SRPASVDG**[S345]**PV**[S348]**PSTNRTHAHRHRGSARL	29	10.3	9.0	3252.5163	362.3980	3092.5837	344.6277
RKRTH**[S527]**AGTSPTITHQKTPSQSSVASIEEY	30	10.0	7.0	3363.6208	481.5245	3283.6545	470.1008
RKRTHSAG**[T530]**SPTITHQKTPSQSSVASIEEY	30	10.0	7.0	3363.6208	481.5245	3283.6545	470.1008
RKRTHSAGT**[S531]**PTITHQKTPSQSSVASIEEY	30	10.0	7.0	3363.6208	481.5245	3283.6545	470.1008
RKRTH**[S527]**AG**[T530]**SPTITHQKTPSQSSVASIEEY	30	10.0	7.0	3443.5872	492.9483	3283.6545	470.1008

RKRTH[S527]AGT[S531]PTITHQKTPSQSSVASIEEY	30	10.0	7.0	3443.5872	492.9483	3283.6545	470.1008
RKRTHSAG[T530][S531]PTITHQKTPSQSSVASIEEY	30	10.0	7.0	3443.5872	492.9483	3283.6545	470.1008
PPEPK[S892]PGEYVNIEF	15	20.0	2.0	1781.7913	891.9029	1701.8250	851.9198
PPEPKSPGE[Y896]VNIEF	15	20.0	2.0	1781.7913	891.9029	1701.8250	851.9198
GSGESSSTRR[S1222]SEDL	15	6.7	3.0	1633.6581	545.5600	1553.6917	518.9045
GSGESSSTRRS[S1223]EDL	15	6.7	3.0	1633.6581	545.5600	1553.6917	518.9045

Chymotrypsin-high specificity							
				Phospho Peptide		Non Phospho Peptide	
Peptide Sequence	Length	% hydro - phobic amino acids	Predicted Charge State(z)	Mono Isotpoic mass	Predicted m/z	Theoretical Mass	Predicted m/z
FVLRAASEAGGPARLE[Y46]YENEKKW	24	20.8	5.0	2863.3694	573.6812	2783.4030	557.6879
RKRTH[S527]AG[T530][S531]PTITHQKTPSQSSVASIEEY	30	10.0	7.0	3523.5535	504.3721	3283.6545	470.1008
KHTQRPGEPEEGARHQHLRLST[S794]SGRLLY	29	13.8	9.0	3419.6960	380.9735	3339.7296	372.0883
GAGAAVGGGGGSSSSSEDVKRHSSA[S1145]F	27	11.1	4.0	2488.0615	623.0227	2408.0952	603.0311
RKRTH[S527]AGTSPTITHQKTPSQSSVASIEEY	30	10.0	7.0	3363.6208	481.5245	3283.6545	470.1008
RKRTHSAG[T530]SPTITHQKTPSQSSVASIEEY	30	10.0	7.0	3363.6208	481.5245	3283.6545	470.1008
RKRTHSAGT[S531]PTITHQKTPSQSSVASIEEY	30	10.0	7.0	3363.6208	481.5245	3283.6545	470.1008
RKRTH[S527]AG[T530]SPTITHQKTPSQSSVASIEEY	30	10.0	7.0	3443.5872	492.9483	3283.6545	470.1008
RKRTH[S527]AGT[S531]PTITHQKTPSQSSVASIEEY	30	10.0	7.0	3443.5872	492.9483	3283.6545	470.1008
RKRTHSAG[T530][S531]PTITHQKTPSQSSVASIEEY	30	10.0	7.0	3443.5872	492.9483	3283.6545	470.1008

Arg-C-proteinase							
				Phospho Peptide		Non Phospho Peptide	
Peptide Sequence	Length	% hydro - phobic amino acids	Predicted Charge State(z)	Mono Isotpoic mass	Predicted m/z	Theoretical Mass	Predicted m/z
LE**[Y46]**YENEKKWR	11	18.2	4.0	1636.7286	410.1894	1556.7623	390.1979
PA**[S341]**VDG**[S345]**PV**[S348]**PSTNR	15	13.3	2.0	1709.6100	855.8123	1469.7110	735.8628
H**[S574]**AFVPTR	8	25.0	3.0	993.4433	332.1550	913.4770	305.4996
LST**[S794]**SGR	7	14.3	2.0	786.3273	394.1709	706.3610	354.1878
VNL**[S1078]**PNR	7	28.6	2.0	878.4011	440.2078	798.4348	400.2247
HS**[S1101]**ETFSSTPSATR	14	7.1	3.0	1573.6410	525.5543	1493.6746	498.8988
HSSA**[S1145]**FENVWLR	12	33.3	3.0	1511.6558	504.8925	1431.6895	478.2371
R**[S1222][S1223]**EDLSAYASISFQKQPEDR	21	14.3	4.0	2573.0836	644.2782	2413.1510	604.2950
PA**[S341]**VDGSPVSPSTNR	15	13.3	2.0	1549.6773	775.8459	1469.7110	735.8628
PASVDG**[S345]**PVSPSTNR	15	13.3	2.0	1549.6773	775.8459	1469.7110	735.8628
PASVDGSPV**[S348]**PSTNR	15	13.3	2.0	1549.6773	775.8459	1469.7110	735.8628
PA**[S341]**VDG**[S345]**PVSPSTNR	15	13.3	2.0	1629.6437	815.8291	1469.7110	735.8628
PA**[S341]**VDGSPV**[S348]**PSTNR	15	13.3	2.0	1629.6437	815.8291	1469.7110	735.8628
PASVDG**[S345]**PV**[S348]**PSTNR	15	13.3	2.0	1629.6437	815.8291	1469.7110	735.8628
R**[S1222]**SEDLSAYASISFQKQPEDR	21	14.3	4.0	2493.1173	624.2866	2413.1510	604.2950
RS**[S1223]**EDLSAYASISFQKQPEDR	21	14.3	4.0	2493.1173	624.2866	2413.1510	604.2950

Clostripain (Clostridiopeptidase B)							
				Phospho Peptide		Non Phospho Peptide	
Peptide Sequence	Length	% hydro - phobic amino acids	Predicted Charge State(z)	Mono Isotpoic mass	Predicted m/z	Theoretical Mass	Predicted m/z
LE**[Y46]**YENEKKWR	11	18.2	4.0	1636.7286	410.1894	1556.7623	390.1979
PA**[S341]**VDG**[S345]**PV**[S348]**PSTNR	15	13.3	2.0	1709.6100	855.8123	1469.7110	735.8628
H**[S574]**AFVPTR	8	25.0	3.0	993.4433	332.1550	913.4770	305.4996
LST**[S794]**SGR	7	14.3	2.0	786.3273	394.1709	706.3610	354.1878
VNL**[S1078]**PNR	7	28.6	2.0	878.4011	440.2078	798.4348	400.2247
HS**[S1101]**ETFSSTPSATR	14	7.1	3.0	1573.6410	525.5543	1493.6746	498.8988
HSSA**[S1145]**FENVWLR	12	33.3	3.0	1511.6558	504.8925	1431.6895	478.2371
R**[S1222][S1223]**EDLSAYASISFQKQPEDR	21	14.3	4.0	2573.0836	644.2782	2413.1510	604.2950
PA**[S341]**VDGSPVSPSTNR	15	13.3	2.0	1549.6773	775.8459	1469.7110	735.8628
PASVDG**[S345]**PVSPSTNR	15	13.3	2.0	1549.6773	775.8459	1469.7110	735.8628
PASVDGSPV**[S348]**PSTNR	15	13.3	2.0	1549.6773	775.8459	1469.7110	735.8628
PA**[S341]**VDG**[S345]**PVSPSTNR	15	13.3	2.0	1629.6437	815.8291	1469.7110	735.8628
PA**[S341]**VDGSPV**[S348]**PSTNR	15	13.3	2.0	1629.6437	815.8291	1469.7110	735.8628
PASVDG**[S345]**PV**[S348]**PSTNR	15	13.3	2.0	1629.6437	815.8291	1469.7110	735.8628
R**[S1222]**SEDLSAYASISFQKQPEDR	21	14.3	4.0	2493.1173	624.2866	2413.1510	604.2950
RS**[S1223]**EDLSAYASISFQKQPEDR	21	14.3	4.0	2493.1173	624.2866	2413.1510	604.2950

Glutamyl endopeptidase							
				Phospho Peptide		Non Phospho Peptide	
Peptide Sequence	Length	% hydro - phobic amino acids	Predicted Charge State(z)	Mono Isotpoic mass	Predicted m/z	Theoretical Mass	Predicted m/z
AGGPARLE[Y46]YE	11	9.1	2.0	1304.5438	653.2792	1224.5775	613.2960
PK[S892]PGE[Y896]VNIE	11	18.2	2.0	1391.5411	696.7778	1231.6084	616.8115
DVKRHSSA[S1145]FE	11	18.2	4.0	1341.5714	336.4001	1261.6051	316.4085
SSSTRR[S1222][S1223]E	9	0.0	3.0	1155.3959	386.1392	995.4632	332.8283
PK[S892]PGEYVNIE	11	18.2	2.0	1311.5748	656.7947	1231.6084	616.8115
PKSPGE[Y896]VNIE	11	18.2	2.0	1311.5748	656.7947	1231.6084	616.8115
SSSTRR[S1222]SE	9	0.0	3.0	1075.4295	359.4838	995.4632	332.8283
SSSTRRS[S1223]E	9	0.0	3.0	1075.4295	359.4838	995.4632	332.8283

LysC							
				Phospho Peptide		Non Phospho Peptide	
Peptide Sequence	Length	% hydro - phobic amino acids	Predicted Charge State(z)	Mono Isotpoic mass	Predicted m/z	Theoretical Mass	Predicted m/z
RFFVLRAASEAGGPARLE[Y46]YENEK	24	20.8	5.0	2852.3646	571.4802	2772.3983	555.4869
RTH[S527]AG[T530][S531]PTITHQK	15	6.7	5.0	1860.7322	373.1537	1620.8332	325.1739
RHSSA[S1145]FENVWLRPGELGGAPK	22	22.7	5.0	2474.1856	495.8444	2394.2192	479.8511
RTH[S527]AGTSPTITHQK	15	6.7	5.0	1700.7995	341.1672	1620.8332	325.1739
RTHSAG[T530]SPTITHQK	15	6.7	5.0	1700.7995	341.1672	1620.8332	325.1739
RTHSAGT[S531]PTITHQK	15	6.7	5.0	1700.7995	341.1672	1620.8332	325.1739
RTH[S527]AG[T530]SPTITHQK	15	6.7	5.0	1780.7659	357.1604	1620.8332	325.1739
RTH[S527]AGT[S531]PTITHQK	15	6.7	5.0	1780.7659	357.1604	1620.8332	325.1739
RTHSAG[T530][S531]PTITHQK	15	6.7	5.0	1780.7659	357.1604	1620.8332	325.1739

Pepsin (pH1.3)							
				Phospho Peptide		Non Phospho Peptide	
Peptide Sequence	Length	% hydro - phobic amino acids	Predicted Charge State(z)	Mono Isotpoic mass	Predicted m/z	Theoretical Mass	Predicted m/z
E**[Y46]**YENEKK	8	0.0	3.0	1181.4642	394.8287	1101.4978	368.1732
PGHRH**[S574]**AFVPTRSYPEEG	18	11.1	5.0	2102.9323	421.5937	2022.9660	405.6005
RLST**[S794]**SGRL	9	22.2	3.0	1055.5125	352.8448	975.5461	326.1893
LLHPPEPK**[S892]**PGE**[Y896]**VNIE	17	23.5	3.0	2077.9162	693.6460	1917.9836	640.3351
FGAGAAVGGGGGSSSSSEDVKRHSSA**[S1145]**F	28	14.3	4.0	2635.1299	659.7898	2555.1636	639.7982
LLHPPEPK**[S892]**PGEYVNIE	17	23.5	3.0	1997.9499	666.9906	1917.9836	640.3351
LLHPPEPKSPGE**[Y896]**VNIE	17	23.5	3.0	1997.9499	666.9906	1917.9836	640.3351

Pepsin (pH1.2)							
				Phospho Peptide		Non Phospho Peptide	
Peptide Sequence	Length	% hydro - phobic amino acids	Predicted Charge State(z)	Mono Isotpoic mass	Predicted m/z	Theoretical Mass	Predicted m/z
E**[Y46]**YENEKKWRHKSSAPKRSIP	21	9.5	8.0	2712.3173	340.0469	2632.3510	330.0511
PGHRH**[S574]**AFVPTRSYPEEG	18	11.1	5.0	2102.9323	421.5937	2022.9660	405.6005
RLST**[S794]**SGRL	9	22.2	3.0	1055.5125	352.8448	975.5461	326.1893
LLHPPEPK**[S892]**PGE**[Y896]**VNIE	17	23.5	3.0	2077.9162	693.6460	1917.9836	640.3351
FGAGAAVGGGGGSSSSSEDVKRHSSA**[S1145]**F	28	14.3	4.0	2635.1299	659.7898	2555.1636	639.7982
LLHPPEPK**[S892]**PGEYVNIE	17	23.5	3.0	1997.9499	666.9906	1917.9836	640.3351
LLHPPEPKSPGE**[Y896]**VNIE	17	23.5	3.0	1997.9499	666.9906	1917.9836	640.3351

Proteinase K							
				Phospho Peptide		Non Phospho Peptide	
Peptide Sequence	Length	% hydro - phobic amino acids	Predicted Charge State(z)	Mono Isotpoic mass	Predicted m/z	Theoretical Mass	Predicted m/z
GGKPG**[S323]**F	7	14.3	2.0	728.2894	365.1520	648.3231	325.1688
DG**[S345]**PV**[S348]**PST	9	11.1	1.0	1005.3093	1006.3166	845.3767	846.3839
NHSR**[S374]**I	6	16.7	3.0	792.3280	265.1166	712.3616	238.4612
G**[T530][S531]**PT	5	0.0	1.0	621.1448	622.1521	461.2122	462.2194
PGHRH**[S574]**A	7	0.0	4.0	840.3392	211.0921	760.3728	191.1005
ST**[S794]**SGRL	7	14.3	2.0	786.3273	394.1709	706.3610	354.1878
PK**[S892]**PGE**[Y896]**V	8	12.5	2.0	1035.3715	518.6930	875.4389	438.7267
NL**[S1078]**PNRNQSA	10	10.0	2.0	1179.5034	590.7590	1099.5370	550.7758
KRHSSA**[S1145]**F	8	12.5	4.0	998.4335	250.6156	918.4672	230.6241
RR**[S1222][S1223]**E	5	0.0	3.0	793.2521	265.4246	633.3194	212.1138
DG**[S345]**PVSPST	9	11.1	1.0	925.3430	926.3503	845.3767	846.3839
DGSPV**[S348]**PST	9	11.1	1.0	925.3430	926.3503	845.3767	846.3839
G**[T530]**SPT	5	0.0	1.0	541.1785	542.1858	461.2122	462.2194
GT**[S531]**PT	5	0.0	1.0	541.1785	542.1858	461.2122	462.2194
PK**[S892]**PGEYV	8	12.5	2.0	955.4052	478.7099	875.4389	438.7267
PKSPGE**[Y896]**V	8	12.5	2.0	955.4052	478.7099	875.4389	438.7267
RR**[S1222]**SE	5	0.0	3.0	713.2858	238.7692	633.3194	212.1138
RRS**[S1223]**E	5	0.0	3.0	713.2858	238.7692	633.3194	212.1138

Staphylococcal peptidase I							
				Phospho Peptide		Non Phospho Peptide	
Peptide Sequence	Length	% hydro - phobic amino acids	Predicted Charge State(z)	Mono Isotpoic mass	Predicted m/z	Theoretical Mass	Predicted m/z
AGGPARLE**[Y46]**YE	11	9.1	2.0	1304.5438	653.2792	1224.5775	613.2960
PK**[S892]**PGE**[Y896]**VNIE	11	18.2	2.0	1391.5411	696.7778	1231.6084	616.8115
DVKRHSSA**[S1145]**FE	11	18.2	4.0	1341.5714	336.4001	1261.6051	316.4085
SSSTRR**[S1222][S1223]**E	9	0.0	3.0	1155.3959	386.1392	995.4632	332.8283
PK**[S892]**PGEYVNIE	11	18.2	2.0	1311.5748	656.7947	1231.6084	616.8115
PKSPGE**[Y896]**VNIE	11	18.2	2.0	1311.5748	656.7947	1231.6084	616.8115
SSSTRR**[S1222]**SE	9	0.0	3.0	1075.4295	359.4838	995.4632	332.8283
SSSTRRS**[S1223]**E	9	0.0	3.0	1075.4295	359.4838	995.4632	332.8283

Thermolysin							
				Phospho Peptide		Non Phospho Peptide	
Peptide Sequence	Length	% hydro - phobic amino acids	Predicted Charge State(z)	Mono Isotpoic mass	Predicted m/z	Theoretical Mass	Predicted m/z
LE**[Y46]**YENEKKWRHKSS	15	13.3	6.0	2075.9466	346.9984	1995.9802	333.6707
LTRRSRTE**[S307]**IT	11	18.2	4.0	1398.6980	350.6818	1318.7317	330.6902
A**[S341]**VDG**[S345]**P	7	14.3	1.0	791.2140	792.2212	631.2813	632.2886
V**[S348]**PSTNRTH	9	11.1	3.0	1077.4604	360.1608	997.4941	333.5053
LNHSR**[S374]**IP	8	25.0	3.0	1002.4648	335.1622	922.4984	308.5068
FRKRTH**[S527]**AG**[T530][S531]**PT	13	7.7	5.0	1684.6525	337.9378	1444.7535	289.9580
LPGHRH**[S574]**AF	9	22.2	4.0	1100.4916	276.1302	1020.5253	256.1386
LST**[S794]**SGR	7	14.3	2.0	786.3273	394.1709	706.3610	354.1878
LLHPPEPK**[S892]**PGE**[Y896]**VN	15	20.0	3.0	1835.7896	612.9371	1675.8569	559.6263
L**[S1078]**PNRNQS	8	12.5	2.0	994.4233	498.2189	914.4570	458.2358
A**[S1145]**FEN	5	20.0	1.0	646.2000	647.2072	566.2336	567.2409
LGSGESSSTRR**[S1222][S1223]**EDLS	17	11.8	3.0	1913.7405	638.9208	1753.8078	585.6099
A**[S341]**VDGSP	7	14.3	1.0	711.2476	712.2549	631.2813	632.2886
ASVDG**[S345]**P	7	14.3	1.0	711.2476	712.2549	631.2813	632.2886
FRKRTH**[S527]**AGTSPT	13	7.7	5.0	1524.7198	305.9512	1444.7535	289.9580
FRKRTHSAG**[T530]**SPT	13	7.7	5.0	1524.7198	305.9512	1444.7535	289.9580
FRKRTHSAGT**[S531]**PT	13	7.7	5.0	1524.7198	305.9512	1444.7535	289.9580
FRKRTH**[S527]**AG**[T530]**SPT	13	7.7	5.0	1604.6861	321.9445	1444.7535	289.9580
FRKRTH**[S527]**AGT**[S531]**PT	13	7.7	5.0	1604.6861	321.9445	1444.7535	289.9580
FRKRTHSAG**[T530][S531]**PT	13	7.7	5.0	1604.6861	321.9445	1444.7535	289.9580
LLHPPEPK**[S892]**PGEYVN	15	20.0	3.0	1755.8233	586.2817	1675.8569	559.6263
LLHPPEPKSPGE**[Y896]**VN	15	20.0	3.0	1755.8233	586.2817	1675.8569	559.6263
LGSGESSSTRR**[S1222]**SEDLS	17	11.8	3.0	1833.7742	612.2653	1753.8078	585.6099
LGSGESSSTRRS**[S1223]**EDLS	17	11.8	3.0	1833.7742	612.2653	1753.8078	585.6099
